# Further assessment of the Genus *Neodon* and the description of a new species from Nepal

**DOI:** 10.1371/journal.pone.0219157

**Published:** 2019-07-17

**Authors:** Nelish Pradhan, Ajay N. Sharma, Adarsh M. Sherchan, Saurav Chhetri, Paliza Shrestha, C. William Kilpatrick

**Affiliations:** 1 Department of Biology, University of Vermont, Burlington, Vermont, United States of America; 2 Center for Molecular Dynamics–Nepal, Kathmandu, Nepal; 3 Department of Biology, Trinity University, San Antonio, Texas, United States of America; 4 Department of Plant and Soil Science, University of Vermont, Burlington, Vermont, United States of America; Universite de Liege, BELGIUM

## Abstract

Recent molecular systematic studies of arvicoline voles of the genera *Neodon*, *Lasiopodomys*, *Phaiomys*, and *Microtus* from Central Asia suggest the inclusion of *Phaiomys leucurus*, *Microtus clarkei*, and *Lasiopodomys fuscus* into *Neodon* and moving *Neodon juldaschi* into *Microtus* (*Blanfordimys*). In addition, three new species of *Neodon* (*N*. *linzhiensis*, *N*. *medogensis*, and *N*. *nyalamensis*) have recently been described from Tibet. Analyses of concatenated mitochondrial (*Cytb*, *COI*) and nuclear (*Ghr*, *Rbp3*) genes recovered *Neodon* as a well-supported monophyletic clade including all the recently described and relocated species. Kimura-2-parameter distance between *Neodon* from western Nepal compared to *N*. *sikimensis* (K2P = 13.1) and *N*. *irene* (K2P = 13.4) was equivalent to genetic distances observed between recognized species of this genus. The specimens sampled from western Nepal were recovered sister to *N*. *sikimensis* in the concatenated analysis. However, analyses conducted exclusively with mitochondrial loci did not support this relationship. The occlusal patterns of the first lower (m1) and third upper (M3) molars were simpler in specimens from western Nepal in comparison to *N*. *sikimensis* from eastern Nepal and India. Twelve craniodental characters and four external field measurements were examined from specimens of *N*. *sikimensis* from eastern Nepal and India, *N*. *irene*, and *Neodon* from western Nepal. *Neodon* from western Nepal were significantly different from *N*. *sikimensis* from eastern Nepal and India in ten out of 16 characters measured and from *N*. *irene* for all characters except ear height. Specimens from western Nepal were smaller in size than *N*. *sikimensis* from Eastern Nepal and India and larger than *N*. *irene*. Together the results of the molecular and morphological analyses indicate that *Neodon* from western Nepal are distinct under the phylogenetic, genetic and morpho species concepts.

## Introduction

The genus *Neodon* (Tribe Arvicolini, Subfamily Arvicolinae) was described by Horsfield [1] and was retained as a genus in early revisions [2–4]. Allen [5] recognized *Neodon* as a subgenus of *Microtus* whereas Ellerman and Morrison-Scott [6] included it in the genus *Pitymys*, a large and rather ill-defined genus with taxa occurring in the Palearctic and Nearctic. Gromov and Polyakov [7] considered *Neodon* a subgenus of *Microtus* whereas Corbet [8] followed the suggestion of Ellerman and Morrison-Scott [6] including *Neodon* in the genus *Pitymys*. Revisions in the 1990’s either recognized *Neodon* as a distinct genus [9, 10] or as a subgenus of *Microtus* [11–13] whereas more recent revisions [14, 15] have recognized as a distinct genus. Central Asia voles including the genera *Neodon*, *Blanfordimys*, and *Phaiomys* have been hypothesized to be Pleistocene relicts due to their primitive molar occlusal patterns resembling the fossil genus *Allophaiomys* [16]. Additionally, *N*. *juldaschi* possesses a karyotype (2n = 54 or 56) thought to be ancestral to the Arvicolini [10, 17].

Musser and Carleton [14] recognized four species of *Neodon* (*forresti*, *irene*, *juldaschi*, *sikimensis*). Several molecular phylogenies have recovered *Neodon* as diphyletic [18–22] with *N*. *juldaschi* in a clade with forms of *Blanfordimys* (recognized either as a distinct genus or as a subgenus of *Microtus*) and forms of *Neodon* other than *N*. *juldaschi* in a clade with *Phaiomys leucurus* [18–22], *Lasiopodomys fuscus* [20], *Microtus clarkei* [18, 21, 22], or *Volemys millicens* [23]. Results from these phylogenetic analyses have led to further changes in the content of the genus with the recognition of *P*. *leucurus* as *N*. *leucurus *[20, 22], *M*. *clarkei* as *N*. *clarkei* [24], *L*. *fuscus* as *N*. *fuscus* [20, 22], and *N*. *juldaschi* as *Microtus* (*Blanfordimys*) *juldaschi* [19, 25]. Three additional species of *Neodon* have been described in recent years from China: *N*. *linzhiensis* [20] and *N*. *nyalamensis* and *N*. *medogensis* [22]. The most recent revision [15] recognized nine species of *Neodon* (*clarkei*, *forresti*, *fuscus*, *irene*, *leucurus*, *linzhiensis*, *medogensis*, *nyalamensis*, and *sikimensis*) while considering *Blanfordimys* as a subgenus of *Microtus* and recognizing *N*. *juldaschi* as *Microtus* (*Blanfordimys*) *juldaschi*.

**Fig 1:**
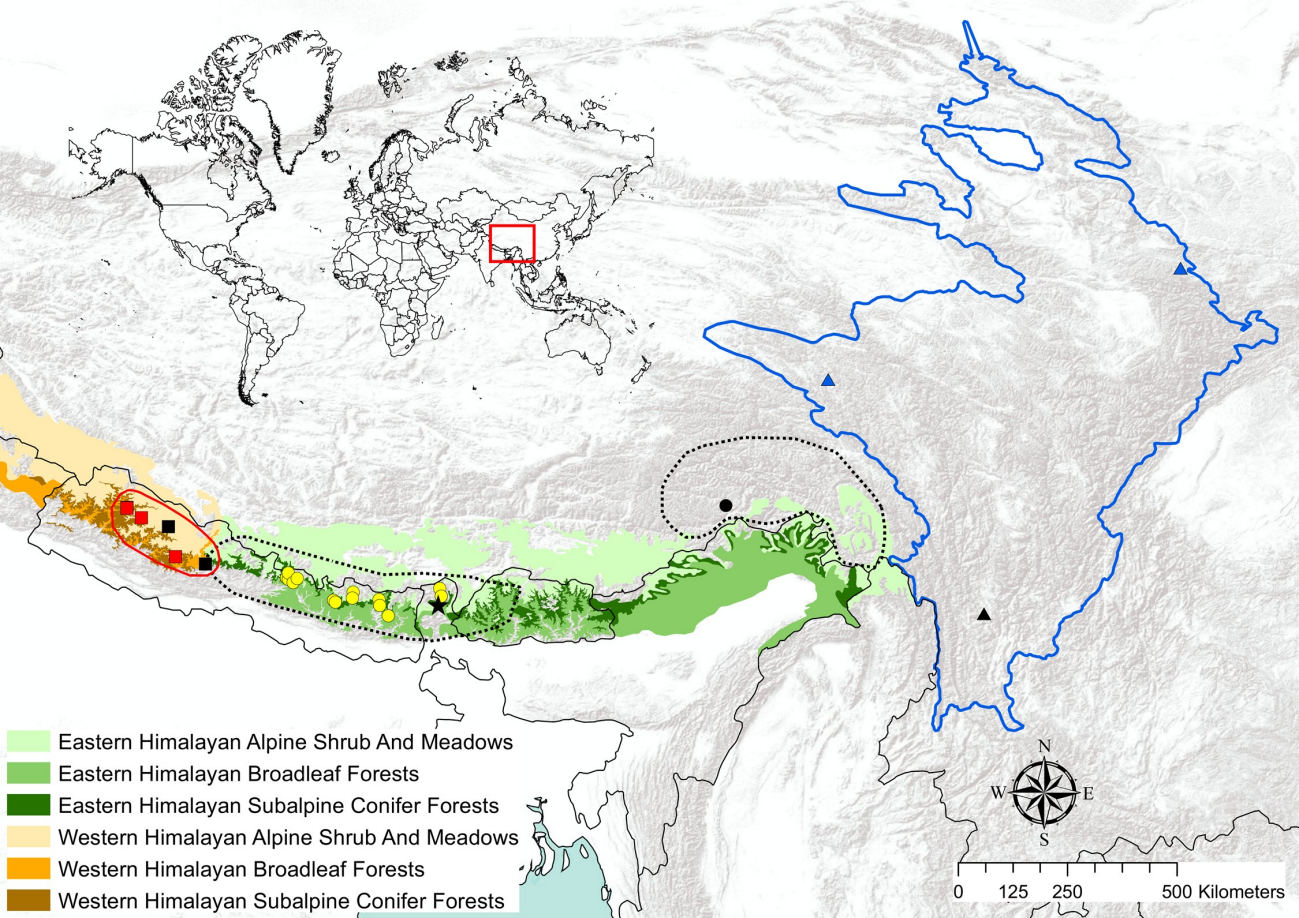
Localities of specimens of *Neodon* examined. Distributional boundaries of *Neodon species novum* (red), *N*. *sikimensis* (dotted), and *N*. *irene* (blue). Collecting localities of museum specimens of *N*. *sikimensis* (circles), *N*. *irene* (triangle), and *N*. *species novum* (squares) examined in the study, with the type locality of *N*. *sikimensis* indicated with a star. Localities of sequenced specimens of *N*. *species novum* (black squares), approximate location of specimens of *N*. *sikimensis* sequenced by Liu et al. [20, 22] (black circle), and *N*. *irene* misidentified as *N*. *sikimensis* in the FMNH (black triangle). Range shapefiles of *N*. *sikimensis* and *N*. *irene* acquired from Terrestrial Mammal dataset IUCN Red List (https://www.iucnredlist.org/resources/spatial-data-download). Shapefiles for Himalayan ecoregions acquired from The Nature Conservancy terrestrial ecoregion data set (http://maps.tnc.org/gis_data.html).

*Neodon sikimensis* occurs on both the western and eastern sides of the Himalayas (Fig 1) with specimens reported from southern Tibet on the eastern side and in Nepal, India, and Bhutan on the western side [14]. In Nepal, the reported distribution of *N*. *sikimensis* extends well into western Nepal [26]. Several specimens from Nepal identified as *N*. *irene* reside at the American Museum of Natural History (AMNH) and the Natural History Museum in London (NHMUK). However, Pearch [26] reclassified specimens at the American Museum of Natural History identified as *N*. *irene* collected from Shey Gompa in western Nepal (AMNH 238055—AMNH 238058, AMNH 238060—AMNH 238063) as *N*. *sikimensis* citing the restricted distribution of *N*. *irene* to China [14, 27]. Specimens at the Natural History Museum in London identified as *N*. *irene* (cataloged as *Pitymys irene forresti*: NHMUK 1954.10.52–1954.10.77) collected from Saipal, Urai Lagna, Garanphu, and Seti in western Nepal could also be excluded from representing *N*. *irene* due to their distribution. Although *N*. *irene* has been included as a synonym of *N*. *sikimensis* by some authors [8, 28, 29], these two species have been distinguished based on differences in dentition and size [30, 31], leading others, including most recent reviews, to treat *N*. *irene* as a distinct species [6, 7, 14, 15]. Nadachowski and Zagorodnyuk [16] observed variation in dental characters among specimens from Nepal great enough to suggest that *N*. *sikimensis* may constitute a group of sibling species. Because of a lack of agreement of the number of species of *Neodon* that occur in Nepal a closer examination is needed.

We report on samples of *Neodon* collected from western Nepal and compare mitochondrial and nuclear DNA sequences obtained with existing sequences from *Neodon* and related species. The objectives of this study are: (1) to compare *Cytb* and other mitochondrial and nuclear sequence data from specimens of *Neodon* to determine if the samples from western Nepal represent a distinct species under the genetic and phylogenetic species concepts; (2) to determine if *Neodon* from western Nepal represent a distinct species under a morphospecies concept by comparison of craniodental measurements and external field measurements with *N*. *sikimensis* from eastern Nepal, India and Tibet, and with *N*. *irene*; and (3) to sample both mitochondrial and nuclear loci to provide better resolution of the phylogenetic relationships between *Neodon* and other genera and subgenera of the Arvicolini.

## Materials and methods

### Specimens and taxonomic sampling

Tissues (ear punches) of *Neodon* specimens (n = 6) from the Myagdi District (Permit no.– 10/070/071) and the Dolpo District (Permit no.– 1/2071/72) of western Nepal were collected in 2013 and 2015 respectively (Table 1). Permits were secured from the Department of National Parks and Wildlife Conservation in Nepal to collect samples from the Nepalese field mouse (*Apodemus gurkha*), at the time considered an endangered species, hence specimens were to be released following the collection of ear punches under the permit conditions. Voucher specimens were prepared for all individuals that perished in the traps overnight (Table 1). Specimens were collected following methods outlined in the American Society of Mammalogists Guidelines [32]. Mitochondrial (*Cytb* and *COI*) and nuclear (*Rbp3* and *Ghr*) sequences for select Arvicolinae and outgroup taxa (*Mesocricetus auratus*, Subfamily Cricetinae) were obtained from GenBank (S1 and S2 Appendices).

**Table 1:** Localities of specimens collected from Nepal with accession numbers of sequences obtained in this study.

Tissue Sample	Voucher	Locality	Coordinates	*Cyt b*	*COI*	*Rbp3*	*Ghr*
GPN16	NP 24	Ghorepani, Myagdi	N 28^o^24.155’ E 83^o^41.865’	MH558123	MH558119	MH558114	MH569055
GPN25	NP 26	Ghorepani, Myagdi	N 28^o^24.077’ E 83^o^41.810’	MH558124	MH558120	MH558115	MH569056
RCH4	na	Rechi, Dolpo	N 29^o^07.035 E 82^o^53.239’	MH558125			MH569057
RGM4	na	Ringmo, Dolpo	N 29^o^10.438’ E 82^o^56.176’	MH558126		MH558116	MH569058
RGM6	na	Ringmo, Dolpo	N 29^o^10.141’ E 82^o^56.413’	MH558127	MH558121	MH558117	MH569059
RGM11	NP44	Ringmo, Dolpo	N 29^o^10.141’ E 82^o^56.413’	MH558128	MH558122	MH558118	MH569060

na = no voucher, specimen released

A total of 170 specimens cataloged as *N*. *sikimensis* from the Field Museum of Natural History (FMNH; n = 168, including a juvenile) and the National Museum of Natural History (NMNH; n = 2), were measured and examined for molar occlusal patterns (S3 Appendix). In addition, 6 specimens cataloged as *N*. *sikimensis* from Sichuan and Yunnan [outside the reported range of *N*. *sikimensis*] provinces in China (east of Tibet) in the FMNH (n = 4) and the NMNH (n = 2) were examined. Measurements were also taken from 8 specimens of *N*. *irene* in the FMNH (n = 2) and the NMNH (n = 6) and from 3 new specimens of *Neodon* collected from western Nepal (S3 Appendix).

### DNA extraction, sequencing, and alignment

DNA extraction and sequencing for *Neodon* sampled in Nepal was carried out at the Center for Molecular Dynamics, Nepal (CMDN). Approximately 25 mg of ear tissue stored in 95% ethanol was used for DNA extractions that were conducted using DNeasy Blood and Tissue Kit (Qiagen, Germany). Tissue samples were air dried for 30 min prior to extraction. Each tissue sample was cut into small pieces (approx. 20) and incubated at 56°C in 180 μl of ATL (Tissue Lysis Buffer) and Proteinase K in a shaking incubator until the tissue samples were completely lysed. DNA was finally eluted in 50 μl of Elution buffer and the quantity and quality of the extracted DNA was assessed on a NanoVue Plus (GE Healthcare Life Sciences) spectrophotometer.

Cytochrome *b* (*Cytb*), cytochrome oxidase I (*COI*), interphotoreceptor retinoid binding protein (*Rbp3*), and growth hormone receptor (*Ghr*) were amplified with the primer pairs: L14274/H15915 [33], COIF/COIR [34], Rbp3217/Rbp31531 [35], and Ghr5/Ghr4 [36] respectively. Amplification of *Cytb* was performed with initial denaturation at 94°C for 5 min followed by 31 cycles of denaturation at 95°C for 1 min, annealing at 50°C for 1 min and extension at 68°C for 1 min and final extension at 68°C for 10 min. Attempts to amplify *Cytb* from “crusties” from six museum specimens included in the morphological analyses were unsuccessful. Amplification of *Rbp3* was carried out with initial denaturation at 94°C for 5 min followed by 35 cycles of denaturation at 94°C for 1 min, annealing at 50°C for 1 min and extension at 68°C for 1.5 min and final extension at 68°C for 10 min. Cycling parameters specified in Galewski et al. [36] were used to amplify *Ghr* whereas *COI* was amplified following the cycling parameters from Zeng et al. [34]. Amplification reactions were conducted in 25 µl volumes (H_2_O – 16.9 μl; 5X PCR buffer+ MgCl_2_−5 μl; 5000 U Taq Polymerase– 0.125 μl; 10 pMol/µl Forward Primer– 0.75 μl; 10 pMol/µl Reverse Primer– 0.75 μl; 10 mM dNTPs– 0.50 μl; DNA template– 1 μl) using OneTaq DNA Polymerase (NEB, Ipswich, MA, USA). PCR products were visualized with ethidium bromide after gel electrophoresis on 2% agarose gels. ExoSAP (Exonuclease and Shrimp Alkaline Phosphatase) (ThermoFisher Scientific) was used to purify the PCR products following manufacturer’s protocol prior to sequencing reactions. Sequencing reactions (10 µl) were conducted with 1 μl of ExoSAP product, 3 µl primer (5 µM/µl), 2 µl nuclease free water and 4 µl of Ready reaction mix containing fluorescent tagged terminator (BigDye v3.1; Applied Biosystems Foster City, CA). The sequencing reaction was further purified using Big Dye X-Terminator Purification Kit (ThermoFisher Scientific) (45 µl SAM solution + 10 µl Big Dye X-terminator solution along with all sequencing PCR product (10 µl) in a 0.5 ml tube. The reactions were optimally diluted in ABI 310 Sequencing Strip tubes for running the final sequencing reaction on ABI 310 avoiding the formation of any air bubbles in the strip tubes.

Chromatograms outputted from the sequencers were visualized and edited in Chromas 2.6.2 (Technelysium: http://technelysium.com.au/wp/chromas/). Sequences from two mitochondrial genes, *Cytb* (1140 bp) and *COI* (1542 bp) and two nuclear genes *Rbp3* (1227 bp) and *Ghr* (869 bp) were aligned using ClustalW [37] in Mesquite 3.50 [38]. The codon positions for each locus were assigned and the alignments of the genes were concatenated in Mesquite. Concatenations for species of *Neodon* were compiled based on sequences from the same specimen or specimens from the same locality when possible (S1 Appendix).

### Genetic distance and phylogenetic analyses

Kimura 2-parameter [39] genetic distances between and within genera were determined from *Cytb* sequences of *Neodon* (S2 Appendix) to compare levels of differentiation observed with values reported for other mammals that have been used to characterize species differentiation under the genetic species concept [40,41]. These measures of genetic differentiation were estimated with MEGA6.0 [42] based on the *Cytb* alignment of the individuals in Table 1 and *Neodon* sequences from GenBank (S2 Appendix).

The concatenated data set was divided into 12 individual partitions *a priori* with each gene (*Cytb*, *COI*, *Rbp3*, and *Ghr*) divided by codon positions. Partition finder 2.1.1 [43] was used to determine the best partitioning scheme and best model for each partition based on the AIC criterion (Table 2) under a likelihood framework using PhyML [44] and the greedy algorithm [45].

**Table 2:** Partitioning scheme and best molecular evolution models selected by PartitionFinder [43].

Data Partitions	Best Model
*Cytb* codon 1	GTR+I+G
*Cytb* codon 2	GTR+I+G
*Cytb* codon 3	GTR+I+G
*COI* codon 1	GTR+I+G
*COI* codon 2	GTR+I
*COI* codon 3	GTR+I+G
*Ghr* codon 1	HKY+G
*Ghr* codon 2	HKY+G
*Rbp3* codon 1	GTR+G
*Rbp3* codon 2	SYM+G
*Rbp3* codon 3 and *Ghr* codon 3	HKY+G

Maximum Likelihood (ML) analysis was conducted with RAxML [46] for the concatenated sequences with 1000 bootstrap replicates using the GTR+I+G model on all partitions shown in Table 2. The majority rule consensus (MRC) tree of the bootstrapped analysis was constructed in Mesquite. A partitioned Bayesian analysis was conducted on the CIPRES portal [47] using the 11 partitions and models in Table 2 for the concatenated dataset in MrBayes 3.2.3 [48]. Two simultaneous runs of 10,000,000 generations with sampling every 1000 generations was carried out. The MrBayes log files for both runs were examined in Tracer 1.6 [49] and a burnin of 2,500,000 generations was set for each run. The runs were combined after discarding the burnin and the MRC tree with posterior probability values was constructed in Mesquite. In addition, partitioned Bayesian analyses of mitochondrial genes (*Cytb* and *COI*), nuclear genes (*Rbp3* and *Ghr*) and each gene were also carried out separately using the models from Table 2 and same parameters as mentioned above.

### Morphometric analyses

**Fig 2:**
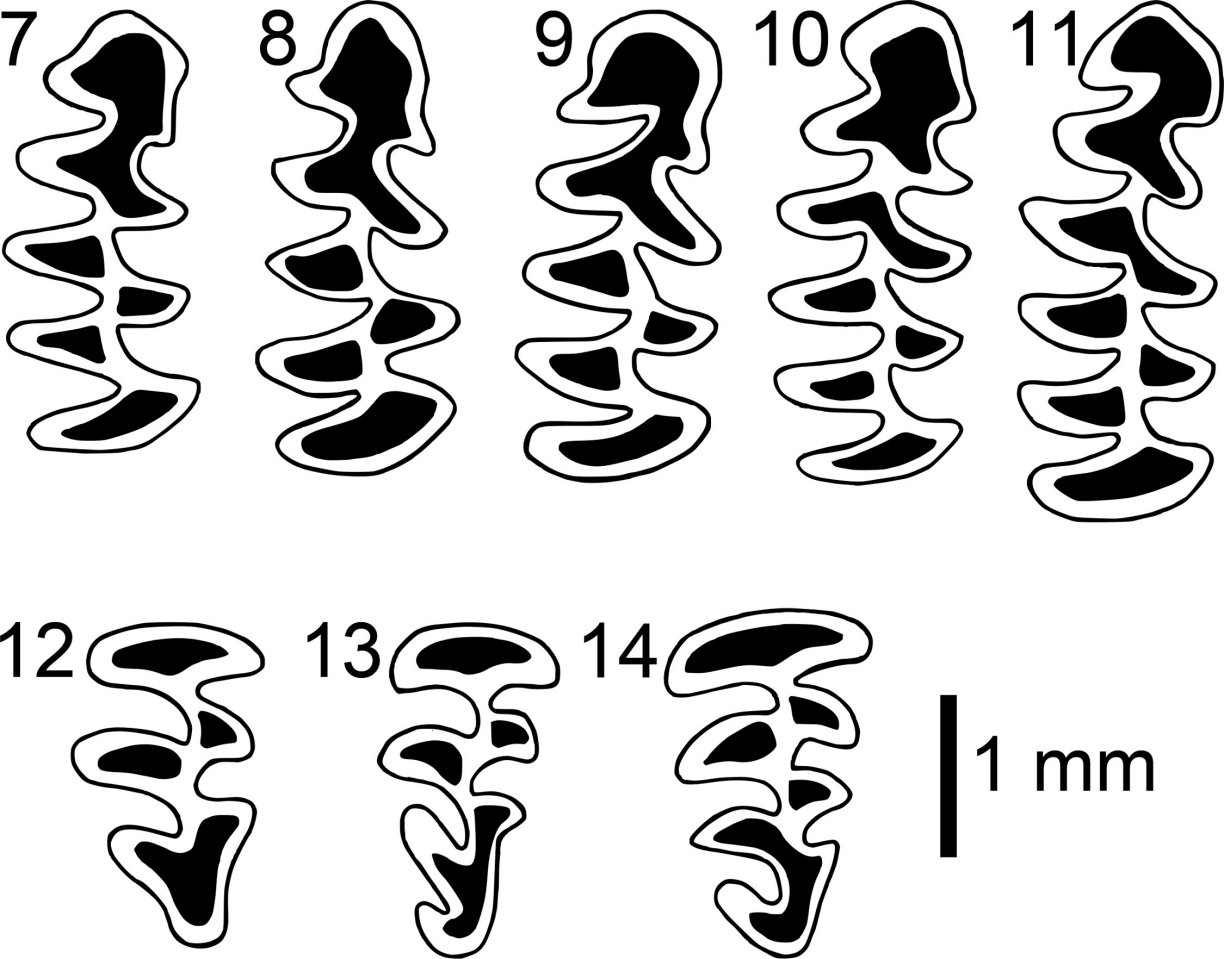
Illustration of occlusal patterns of m1 (top) and M3 (bottom) molars of *Neodon*. Illustrations derived from photos of the dentition of specimens (S1 Fig) examined by Nadachowski and Zagorodnyuk following their numbering system (see Fig 2 [16]).

From examination of photos (S1 Fig) of the dentition of the specimens used by Nadachowski and Zagorodnyuk (see Fig 2 [16]) to illustrate the variation observed in the occlusal pattern of the first lower molar (m1) and third upper (M3) molar among specimens of *N*. *sikimensis*, diagrams of the discrete patterns of the m1 and M3 were constructed (Fig 2). The numerical designations of the original illustration (see Fig 2 [16]) were used as code for the patterns observed. The occlusal patterns of the first lower molar varied from a tooth with an anterior loop followed by one pair of confluent triangles followed by three alternating closed triangles and a posterior loop resulting in four lingual and three labial folds (Fig 2, pattern 7) to a tooth with an anterior loop followed by two pairs of confluent triangles followed by three alternating closed triangles and a posterior loop resulting in five lingual and four labial folds (Fig 2, pattern 11). Patterns 8–10 (Fig 2) vary in the degree of development of the second pair of confluent triangles. The upper third molar varied from a tooth with an anterior loop followed by three alternating triangles, the first two which were closed and a third which was either opened or closed, and a posterior loop without a medially inflected end resulting in two lingual folds (Fig 2, pattern 12) to a tooth with and anterior loop followed by three alternating closed triangles and a posterior loop with a clearly defined medially inflected end resulting in three lingual folds (Fig 2, pattern 14). Pattern 13 (Fig 2) has three closed triangles and a weakly defined medially inflected end of the posterior loop.

Discrete numerical codes were assigned to the m1 and M3 molar patterns of 177 specimens of *Neodon* examined (S3 Appendix). Variation in the distribution of occlusal patterns was examined from a geographic prospective.

Eight cranial characters were measured (±0.01 mm) with dial calipers including: skull greatest length (SGL), skull basal length (SBL), condylobasal length (CBL), zygomatic breadth (ZB), mastoidal breadth (MB), least interorbital width (IOW), skull height (SH), and auditory bullae length (ABL). In addition, four dental characters were also measured including: length of maxillary tooth-row (LMxT), greatest width across upper molars (MM), length of the mandibular tooth-row (LMbT), and breadth across the two upper incisors (TUIB). External measurements (in mm) including total body length (TBL), tail length (TL), length of hind foot (HF), and ear length (EL) were obtained from skin tags on the specimens. Measurements for the three voucher specimens housed in Nepal are provided in S1 Table.

Estimation of descriptive statistics (mean, range, and standard error) of all measurements for each species (S3 Appendix) and one-way ANOVA with pairwise (t-test) comparisons between *N*. *species novum* (n = 15), *N*. *sikimensis* (n = 157 excluding the juvenile FMNH 142100), and *N*. *irene* (n = 14) were conducted on JMP Pro 13.0. Individuals of *N*. *irene* examined include specimens FMNH 33939–33942, NMNH 240368 and NMNH 259931 which are cataloged as *N*. *sikimensis* but were collected outside of the currently recognized range of *N*. *sikimensis* and displayed molar occlusal patterns characteristic of *N*. *irene*. Individuals with missing measurements (S3 Appendix) and FMNH 142100 (juvenile) were excluded from the dataset and measurements were log transformed before conducting multivariate analyses. Statistical significance of differences between *N*. *species novum* (n = 12), *N*. *sikimensis* (n = 74), and *N*. *irene* (n = 8) were tested using multivariate analysis of variance (MANOVA) with Hotelling's pairwise comparisons on the log transformed measurements where *p*-values are reported as Bonferroni-corrected *p*-values. Canonical variate analysis (CVA) of *N*. *species novum* (n = 12), *N*. *sikimensis* (n = 74), and *N*. *irene* (n = 8) was performed to visualize separation among the species. Variable loadings used in the CVA were based on the product of correlated coefficients of principal components of the log transformed measurements. A scatter plot displaying the first two canonical axes was plotted along with convex hulls, where the axes represent linear combinations of the original variables in a Principal Component Analysis. Both the MANOVA and CVA were performed in the statistical package PAST [50].

### Nomenclatural acts

The electronic edition of this article conforms with the requirements of the amended Code of Zoological Nomenclature, and hence the new name contained herein are available under that Code from the electronic edition of this article. This published work and the nomenclatural acts it contains have been registered in ZooBank, the online registration system for the ICZN. The ZooBank LSIDs (Life Science Identifiers) can be resolved and the associated information viewed through any standard web browser by appending the LSID to the prefix http://zoobank.org/. The LSID for this publication is urn:lsid:zoobank.org:pub: E296Ø27B-E533-4DF7-9720-BFFDBC5FB48D. The electronic edition of this work was published in a journal with an ISSN and has been archived and is available from the following digital repositories: PubMed and LOCKSS.

## Results

### Cytochrome *b* divergence within *Neodon*

Kimura 2-parameter (K2P) genetic distances between specimens of *Neodon* sequenced from wester Nepal and *N*. *sikimensis* (mean = 13.1) or *N*. *irene* (mean = 13.4) were comparable to the K2P distances between recognized species of *Neodon* (mean = 11.1–14.4) (Table 3). Large genetic distances between the western Nepal *Neodon* sampled from Dolpo and Myagdi Districts, Nepal were also observed (mean = 7.8; range = 7.6–8.0) (Table 4).

**Table 3:** Mean cytochrome *b* Kimura 2-parameter distance (percent) within and between species of *Neodon*.

Species		Within Species	Between Species							
	n		*Nsik*	*Ncla*	*Nfus*	*Nire*	*Nleu*	*Nlin*	*Nmed*	*Nnya*
Western Nepal	6	4.3	13.1	14.7	14.9	13.4	12.5	14.6	13.0	14.0
*N*. *sikimensis*	11	5.6		12.0	14.4	12.2	11.8	12.5	11.1	11.1
*N*. *clarkei*^a^	3	0.4			13.3	13.3	12.6	13.7	11.6	12.2
*N*. *fuscus*	16	0.6				12.5	13.2	14.5	14.5	13.1
*N*. *irene*	70	4.0					11.6	12.2	12.0	11.7
*N*. *leucurus*^b^	5	2.0						13.5	13.4	12.6
*N*. *linzhiensis*	4	0.7							12.8	13.1
*N*. *medogensis*	5	0.4								12.9
*N*. *nyalamensis*	5	0.2								

^a^ *N*. *clarkei* AY641526 not included

^b^ *N*. *leucurus* AM392371 not included

**Table 4:** Cytochrome *b* Kimura 2-parameter distances (percent) between samples from western Nepal.

Specimen	District	GPN25	RCH4	RGM4	RGM6	RGM11
GPN16	Myagdi	0.5	7.6	7.7	7.6	7.7
GPN25	Myagdi		7.9	8.0	7.9	8.0
RCH4	Dolpo			0.1	0.0	0.3
RGM4	Dolpo				0.1	0.4
RGM6	Dolpo					0.3
RGM11	Dolpo					

### Phylogenetic analyses

**Fig 3:**
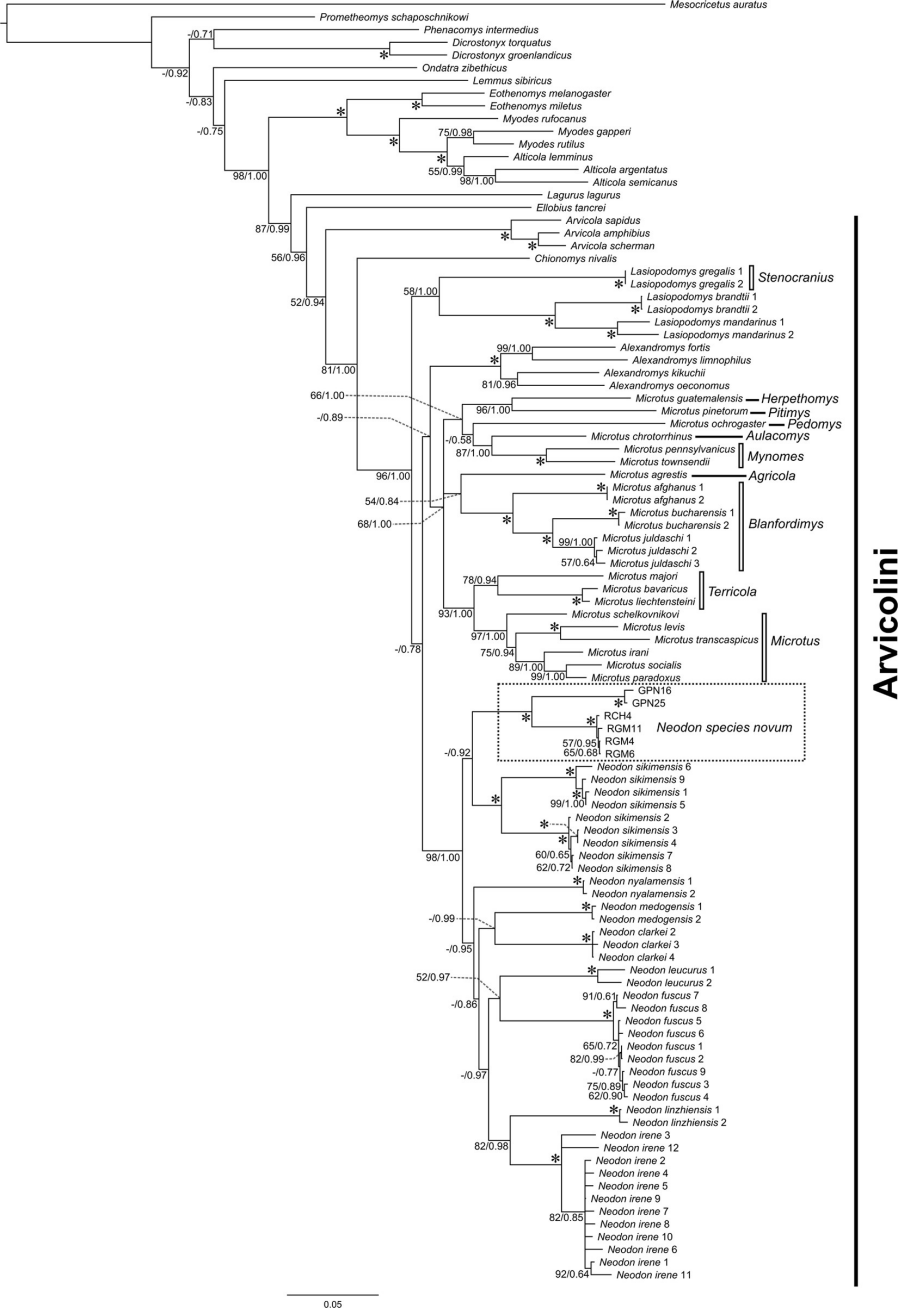
Arvicolinae phylogenetic tree. Bayesian tree of concatenated *Cytb*, *COI*, *Rbp*3, and *Ghr* genes with nodal support as Maximum Likelihood bootstraps (1000 replicates) and Bayesian posterior probability values (ML/PP; only if >50%) where an asterisk (*) refers to ML = 100 and PP = 1.00.

*Neodon* formed a strongly supported clade in both the Maximum Likelihood (ML = 99) and Bayesian analyses (PP = 1.00) using the concatenated dataset of four genes (Fig 3). Monophyly of *Neodon* was supported by all loci: concatenated analyses of mitochondrial (PP = 1.00; S2 Fig) and nuclear loci (PP = 1.00; S3 Fig) as well as *Cytb* (PP = 1.00; S4 Fig), *COI* (PP = 1.00; S5 Fig), *Rbp3* (PP = 1.00; S6 Fig), and *Ghr* (PP = 1.00; S7 Fig) gene trees. *Neodon* was recovered sister to a clade containing *Microtus* and *Alexandromys* (ML<50; PP = 0.78) with the four-gene concatenated dataset.

Specimens of *Neodon* from western Nepal (*N*. *species novum*) formed a well-supported clade in the concatenated analyses (ML = 100; PP = 1.00; Fig 3), mitochondrial loci (PP = 1.00; S2 Fig), nuclear loci (PP = 1.00; S3 Fig), and all individual gene trees (PP = 1.00; S4–S7 Figs). The *N*. *species novum* clade was sister to *N*. *sikimensis* (ML<50; PP = 0.92) with the four-gene concatenated dataset, however this relationship was not recovered in trees built with only mitochondrial loci, nuclear loci alone, or individual genes. *Neodon species novum*, *N*. *leucurus*, and *N*. *fuscus* formed a weakly supported clade with the concatenated mitochondrial loci (PP = 0.66; S2 Fig). Sister species relationships between *irene/linzhiensis* (ML = 82; PP = 0.98), *leucurus/fuscus* (ML = 52; PP = 0.97), were recovered with both likelihood and Bayesian analyses, whereas *medogensis*/*clarkei* was supported only in the Bayesian analyses (PP = 0.99) (Fig 3). The *species novum/sikimensis* clade recovered in the analysis of the concatenated dataset was sister to a clade containing the other 7 species of *Neodon* examined in this study (Fig 3).

The taxon, previously recognized as *N*. *juldaschi*, was recovered in a clade with taxa of *Microtus* of the subgenus *Blanfordimys* (ML = 100; PP = 1.00; Fig 3) with the four-gene concatenated dataset. This relationship was well supported in trees built with individual genes (S4, S6 and S7 Figs) other than *COI* (S5 Fig) for which no sequence data are available for *juldaschi*. A sister taxon relationship was recovered between the well supported subgenus *Blanfordimys* clade and *Microtus* (*Agricola*) *agrestis* from the four-gene concatenated dataset (ML = 54; PP = 0.84; Fig 3) and the *Cytb* data (PP = 0.99; S4 Fig). However, the subgenus *Blanfordimys* clade was recovered as sister to the *Neodon* clade in the analysis of the *Ghr* data (PP = 0.88; S7 Fig).

*Neodon* was recovered as the sister taxon to a clade containing the genera *Microtus* and *Alexandromys* from analysis of the four-gene concatenated dataset, however, this relationship was poorly supported (ML<50; PP = 0.70; Fig 3). Other sister taxon relationships recovered include a *Microtus* clade from analysis of only mitochondrial genes (PP = 0.57; S2 Fig), an *Alexandromys* clade from analysis of only *Cytb* data (PP = 0.90; S4 Fig), and a *Blanfordimys* clade from the analysis of the *Rbp3* data (PP = 0.88; S6 Fig).

### Molar occlusal patterns

The new specimens of *Neodon* collected from western Nepal had occlusal patterns of their m1 (S8 Fig) that were best characterized as discrete pattern 9 (Fig 2) and of their M3 that were best characterized as patterns 13 (n = 1) or 14 (n = 2). These patterns were simpler than the patterns observed in a paratype of *N*. *sikimensis* and specimens from *N*. *sikimensis* reported from Tibet [20] or examined from Sikkim (Table 5). Occlusal patterns were more variable among specimens cataloged as *N*. *sikimensis* from Nepal with both the m1 and M3 varying across the range of patterns identified by Nadachowski and Zagorodnyuk [16]. However, more complex patterns (m1 –patterns 10 & 11 and M3 –patterns 13 & 14) were observed in specimens in eastern Nepal whereas simpler patterns (m1 –patterns 7–9 and M3 –patterns 12 &13) were characteristic of specimens from western Nepal (Table 5). Based on the observed geographic variation of occlusal patterns in specimens from Nepal, we hypothesize that the specimens from western Nepal, including the newly collected material that are genetically distinct from *N*. *sikimensis*, represent a distinct species based on morphological species concept.

**Table 5:** Occlusal patterns of m1 and M3 in specimens of *Neodon* examined.

	m1 occlusal patterns					M3 occlusal patterns		
**Specimens from Nepal**	**7**	**8**	**9**	**10**	**11**	**12**	**13**	**14**
New specimens (S8 Fig)			3			2	1	
Eastern Nepal		1	1	25	111	3	21	114
Western Nepal	1	11				7	5	
**Specimens outside of Nepal**								
*N*. *sikimensis* paratype				*				*
*N*. *sikimensis* Tibet [20]					*			*
*N*. *sikimensis* from Sikkim					18		3	15
Sichuan and Yunnan specimens cataloged as *N*. *sikimensis*	6					6		
*N*. *irene* holotype	*					*		

* pattern observed in type specimens or reported in literature

Specimens from Sichuan and Yunnan catalogued as *N*. *sikimensis* had m1 occlusal patterns assigned to pattern 7 (Fig 2) and M3 occlusal patterns assigned to pattern 12, identical to some of the patterns reported for *N*. *irene* from Sichuan [16]. A few populations of *Neodon* examined from northeastern Nepal, Khumjung, Num and Balutar (S9 Fig), were observed to have molar with rather simple occlusal patterns (m1 –patterns 8–10; M3 –pattern 12).

### Morphometric analyses

Pairwise t-tests showed that specimens in the FMNH from Sichuan and Yunnan catalogued as *N*. *sikimensis* were only significantly differenty from *N*. *irene* for two characters (LMxT and LMbT), whereas they differed from *N*. *sikimensis* by all characters except MB. The specimens catalogued as *N*. *sikimensis* from Sichuan and Yunnan were pooled with *N*. *irene* for all subsequent statistical analyses.

**Fig 4:**
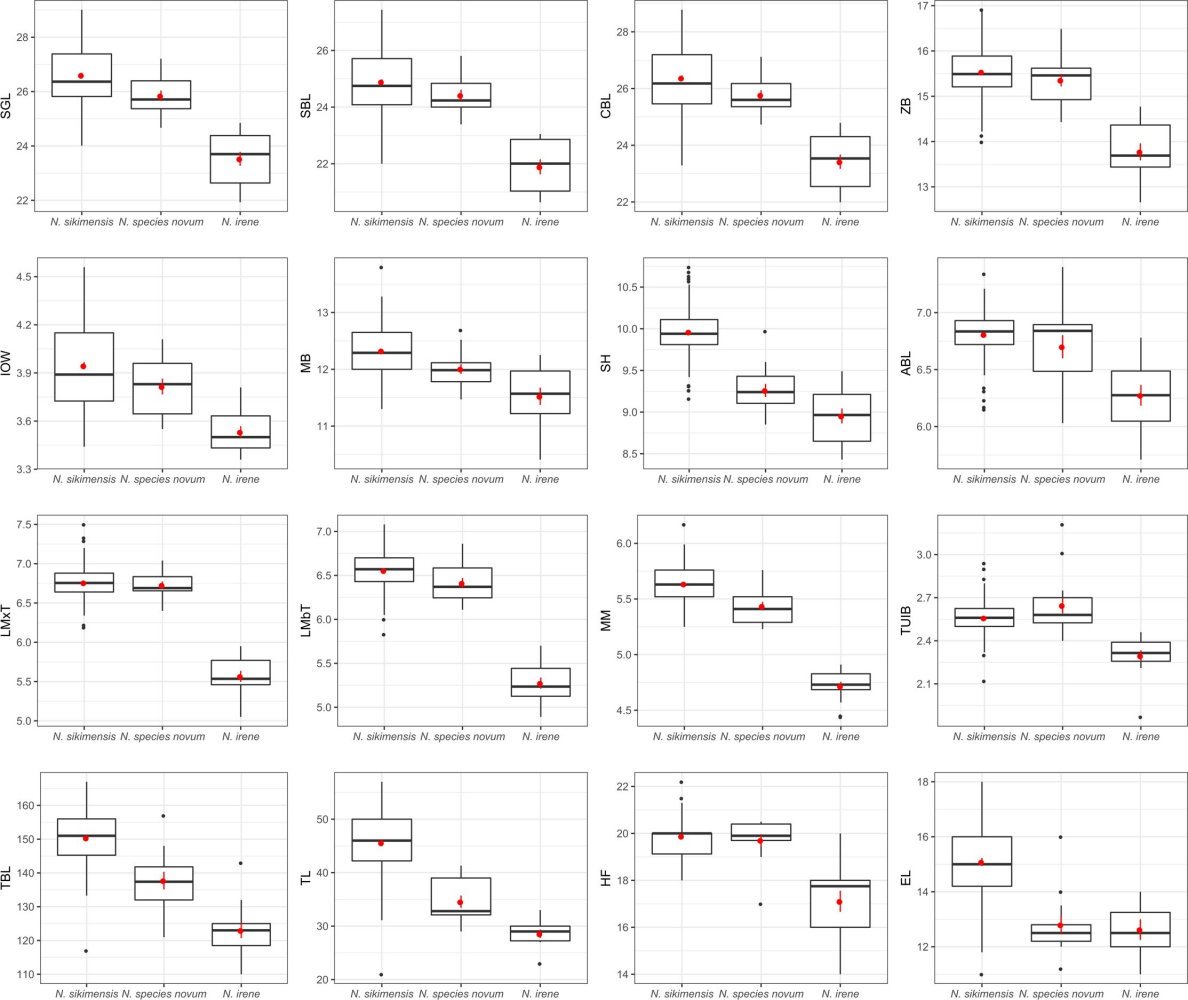
Box plots of morphometric characters. Box plots for eight cranial, four dental, and four external field measurements comparing specimens of *Neodon species novum* (n = 15), *N*. *sikimensis* (n = 146), and *N*. *irene* (n = 14). Mean±SEM in red and median denoted by thick black horizontal bar. SGL = skull greatest length, SBL = skull basal length, CBL = condylobasal length, ZB = zygomatic breadth, MB = mastoid breadth, IOW = least interorbital width, SH = skull height, ABL = auditory bullae length, LMxT = length of maxillary tooth row, LMbT = length of mandibular tooth row, MM = width across upper molars, TUIB = breath across upper incisors, TBL = total body length, TL = tail length, HF = length of hind foot, and EL = length of ear.

One-way ANOVA comparing *Neodon species novum*, *N*. *sikimensis*, and *N*. *irene* resulted in statistically significant (*p*<0.0001) differences between the species for all measurements (Table 6). *Neodon species novum* from western Nepal were significantly smaller than *N*. *sikimensis* and larger than *N*. *irene* (Fig 4 and Table 6). ANOVA indicated significant differences among the three species (*N*. *species novum*, *N*. *sikimensis*, and *N*. *irene*) for all observed characters (p<0.0001: Table 6). Post hoc pairwise comparisons using a t-test between the species revealed that *N*. *species novum* differed from *N*. *sikimensis* and *N*. *irene* with 10 and 15 characters out of 16 respectively (Table 6).

**Table 6:** Summary of one-way ANOVA and pairwise t-test comparison between *Neodon species novum* (Nsn), *N*. *sikimensis* (Ns), and *N*. *irene* (Ni).

	*N*. *species novum*		*N*. *sikimensis*		*N*. *irene*		One-way ANOVA		Pairwise Differences (*p*-values)		
Measurement	Mean±SE	n	Mean±SE	n	Mean±SE	n	F ratio	p-value	Nsn-Ns	Nsn-Ni	Ns-Ni
SGL	25.85±0.19	15	26.60±0.09	140	23.53±0.26	14	56.9128	<0.0001	0.0083	<0.0001	<0.0001
SBL	24.42±0.20	14	24.89±0.09	139	21.89±0.27	12	48.0068	<0.0001	0.1	<0.0001	<0.0001
CBL	25.77±0.17	14	26.37±0.09	139	23.42±0.25	14	48.2854	<0.0001	0.0478	<0.0001	<0.0001
ZB	15.36±0.14	15	15.54±0.05	144	13.77±0.19	14	56.5921	<0.0001	0.2638	<0.0001	<0.0001
IOW	3.82±0.05	15	3.95±0.02	151	3.53±0.04	14	17.3427	<0.0001	0.0653	0.0035	<0.0001
MB	12.01±0.09	14	12.32±0.04	141	11.52±0.15	13	21.3895	<0.0001	0.0112	0.0055	<0.0001
SH	9.26±0.08	15	9.96±0.02	141	8.95±0.09	14	103.2817	<0.0001	<0.0001	0.006	<0.0001
ABL	6.70±0.10	15	6.81±0.02	148	6.27±0.09	14	35.809	<0.0001	0.0819	<0.0001	<0.0001
LMxT	6.73±0.05	15	6.76±0.02	152	5.57±0.07	14	199.3485	<0.0001	0.5923	<0.0001	<0.0001
LMbT	6.41±0.06	15	6.56±0.02	153	5.27±0.06	14	217.4154	<0.0001	0.0174	<0.0001	<0.0001
MM	5.44±0.04	15	5.64±0.01	153	4.71±0.04	14	188.8733	<0.0001	<0.0001	<0.0001	<0.0001
TUIB	2.65±0.06	15	2.56±0.01	151	2.30±0.04	14	31.2322	<0.0001	0.0147	<0.0001	<0.0001
TBL	137.73±2.62	13	150.37±0.84	90	123.00±2.29	14	73.8475	<0.0001	<0.0001	<0.0001	<0.0001
TL	34.56±1.16	13	45.59±0.63	90	28.57±0.80	14	71.5869	<0.0001	<0.0001	0.0059	<0.0001
HF	19.71±0.26	13	19.88±0.08	90	17.11±0.45	14	52.5545	<0.0001	0.5347	<0.0001	<0.0001
EL	12.80±0.33	13	15.08±0.15	89	12.63±0.38	8	25.6296	<0.0001	<0.0001	0.7741	<0.0001

**Fig 5:**
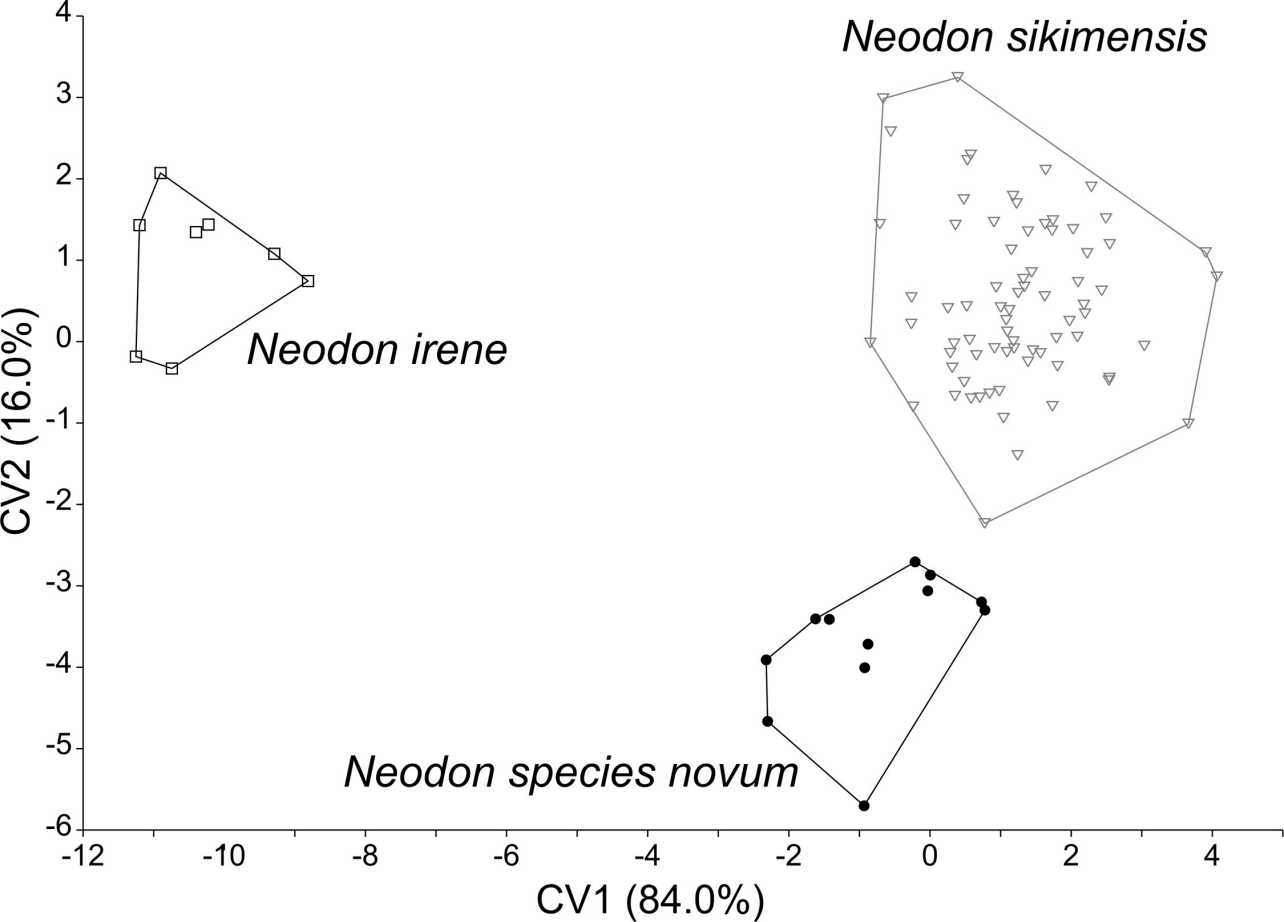
Projection of canonical variates from discriminant analyses. Includes analysis of 8 cranial (SGL, SBL, CBL, ZB, MB, IOW, SH, and ABL), 4 dental (LMxT, LMbT, M-M, and TUIB) and 4 external field measurements (TBL, TL, HF, and EL) for *Neodon species novum* (n = 12), *N*. *sikimensis* (n = 74), and *N*. *irene* (n = 8).

MANOVA results found the three species differed significantly from each other (Wilks’ λ = 0.02794; F = 23.67; *p* < 0.0001). Hotelling’s *P* indicated that *N*. *sikimensis* differed significantly from *N*. *species novum* (*p* = 4.63E-13) and *N*. *irene* (*p* = 1.27E-29), whereas *N*. *species novum* and *N*. *irene* did not (*p* = 0.27). MANOVA comparing just *N*. *species novum* and *N*. *irene*, however, showed significant difference between the two groups (Wilks’ λ = 0.00327; F = 57.2; *p* = 0.0033). Discriminant analysis or CVA clearly separated *N*. *irene* from *N*. *species novum* and *N*. *sikimensis* with CV1 separating *N*. *irene* from *N*. *sikimensis* and *N*. *species novum*, while CV2 separated *N*. *sikimensis* and *N*. *species novum* (Fig 5). Specimens classified with the discriminant analysis based on the Mahalanobis distance and cross-validated with a jackknifing procedure confirmed species were correctly identified in 97.87% of cases, incorrectly classifying 2 out 74 *N*. *sikimensis* specimens (FMNH 104299 and FMNH 142098) as *N*. *species novum*.

## Discussion

### *Neodon* of Western Nepal

Presently two species of *Neodon* are recognized as occurring in Nepal, *N*. *sikimensis* and *N*. *leucurus* [26], though the molar patterns of two specimens (NHML 541076 & NHML 662583) referred to as *N*. *irene* were illustrated by Nadachowski and Zagorodnyuk [16]. In addition, specimens cataloged as *N*. *irene* collected from locations in Nepal are housed in the American Museum of Natural History and the Natural History Museum of London. Pearch [26] included some of these specimens reported as *N*. *irene* as *N*. *sikimensis* based on the range of *N*. *irene* being restricted to China [14, 27] rather than examination of these specimens. The molar occlusal patterns of the specimens *N*. *irene* from Nepal illustrated by Nadachowski and Zagorodnyuk (see Fig 3 [16]) are very similar to the m1 pattern 9 and the M3 patterns 12 and 13 presented for *N*. *sikimensis* (see Fig 2 [16]).

Sequence data obtained from specimens on *Neodon* from western Nepal demonstrated considerable genetic differentiation (*Cytb* K2P) from *N*. *sikimensis*, *N*. *irene*, *N*. *leucurus*, and other species of *Neodon*. Mean genetic distance (K2P) of *Cytb* between *N*. *sikimensis* and the *N*. *species novum* (13.1) exceeds the K2P values calculated between *N*. *sikimensis* and all other species of *Neodon* (range = 11.1–12.5) except for *N*. *fuscus* (14.4) and is comparable to genetic distances between recognized species of *Neodon* (range = 11.1–14.9) (Table 3). Moreover, the mean genetic distance derived between *N*. *sikimensis* and *N*. *species novum* (K2P = 13.1) is larger than the K2P distances reported within Rodentia for sister species (mean K2P = 7.3, range = 1.3–13.0) and comparable to intrageneric distances (mean K2P = 10.9, range = 4.9–16.9) for species of rodents [41]. In a comparable “genus”, *Microtus*, the reported mean K2P distances of *Cytb* was 6.8 (range = 4.3–11.1) between sister species and 10.9 (range = 5.1–12.8) for congeneric species [41], both of which are smaller than what was observed between *N*. *sikimensis* and the *Neodon* from western Nepal. Hence, *N*. *species novum* should be considered a distinct species under the genetic species concept based of the large genetic distances (*Cytb* K2P) between it and other species of the genus.

### Systematics of *Neodon*

Hinton [2] provided a description of the essential features of the genus *Neodon* and commented on the presence of three closed triangles on the m1 as in *Pitymys*, instead of the five, characteristic of *Microtus*. *Neodon* is not as highly modified for subterranean habits as *Pitymys*, with moderately long ears clearly visible above the fur [2].

Our molecular analyses support the conclusion of other recent studies [20–22] and revisions [14, 15] in the recognition of *Neodon* as a distinct genus of the Arvicolini. Unlike previous molecular studies where the *Neodon* clade was recovered as a monophyletic clade within the *Microtus* clade [20–22], it was recovered in our phylogenetic analysis as the sister group to a *Microtus-Alexandromys* clade though with rather low support. Furthermore, our molecular phylogeny supports the conclusion regarding the taxonomic treatment of *M*. (*Blanfordimys*) *juldashi* [15, 19, 25] rather than retaining it in the genus *Neodon* [14].

Several misplaced sequences were initially detected in our phylogeny, including sequences identified as *M*. *clarkei* (AY641526) from Luo et al. [51] and *N*. *leucurus* (AM392371 and AM392394) from Galewski et al. [36] which appear to represent misidentified specimens of *N*. *irene* and *N*. *fuscus* respectively (represented as *N*. *irene* 12 and *N*. *fuscus* 9 in Fig 3). The *Cytb* sequence AY641526 was originally placed in GenBank by Luo et al. [51] as from *M*. *clarkei* but identified as *N*. *irene* (though included in their appendix as *M*. *clarkei*) by Liu et al. [20, 22]. This sequence (AY641526) has a 95% identity with *Cytb* sequences from *N*. *irene* and only an 88% identity with sequences from *M*. *clarkei*, thus we conclude this sequence is representative of *N*. *irene*. Similarly, *Cytb* sequence (AM392371 and *Ghr* sequence AM392394 were submitted to GenBank by Galewski et al. [36] as sequences from *N*. *leucurus* but changed to *Lemmus leucurus* in Chen et al. [23] and to *N*. *fuscus* in Liu et al. [22]. The *Cytb* sequence AM392371 has a 99% identity with *Cytb* sequences from *N*. *fuscus* and an 89% identity with *N*. *leucurus* sequences. In addition, the *Rbp3* sequence AY163593 submitted by Weksler [52] as from a *N*. *sikimensis* was derived from specimen USNM449126 which is cataloged as *N*. *irene* from Qinghai, China. This sequence (AY163593) was concatenated with the *Gh*r sequence AY294924 from a specimen (USNM449173) of *N*. *irene* collected from the same location in our analysis (*N*. *irene* 6). The *Rbp3* sequence AM919400 was excluded from our concatenated analyses due to uncertainty as whether it was derived from *N*. *fuscus* or *N*. *irene* but was included in the *Rbp*3 gene tree (S6 Fig). Arvicolines are often difficult to distinguish morphologically, subsequently leading to misidentification of the taxon that was the source of molecular data. Unfortunately, no voucher specimens are associated with these sequences from misidentified specimens other than AY163593. This further demonstrates the importance of voucher specimens and the identification of those vouchers in association with sequences in GenBank and in the publications reporting new sequence data. In addition, once these misidentifications are recognized the database (GenBank) needs to be corrected. Without correction of the taxon misidentification in the database the use of these misidentified sequences will continue to cause confusion in the literature and contribute further to the confusion in resolving the phylogeny of an already problematic group.

All species of *Neodon*, including *N*. *species novum*, form strong supported clades (ML = 100; PP = 1.00) that are reciprocally monophyletic with other *Neodon* clades. This well support reciprocal monophyly with other clades of *Neodon*, supports the recognition of *N*. *species novum* as a distinct species under a phylogenetic species concept [53, 54]. The phylogeny (Fig 2) supports the currently recognized content of the genus [15] with the addition of *N*. *species novum* and presents greater resolution of the relationships among *Neodon* species (excluding *N*. *forresti* for which no data are available). However, there are discrepancies among relationships recovered within *Neodon* between mitochondrial and nuclear loci. All phylogenies show a distant relationship between *N*. *irene* and either *N*. *species novum* or *N*. *sikimensis*. Phylogenies recovered from different concatenation of genes (mitochondrial+nuclear or mitochondrial and nuclear separately) differ in the placement of *Neodon* specimens from western Nepal. Use of concatenated mitochondrial+nuclear loci recovers *N*. *species novum* sister to *N*. *sikimensis*, whereas use of just mitochondrial loci recovers *N*. *species novum* in a weakly supported clade containing *N*. *leucurus*, and *N*. *fuscus* and the use of nuclear loci exclusively does not resolve the *N*. *species novum/N*. *sikimensis* relationship. The use of mitochondrial loci to construct phylogenies for arvicolines has been shown to yield low support values and polytomies among the deeper nodes [36, 55]. Most *Neodon* species (*N*. *clarkei*, *N*. *leucurus*, *N*. *linzhiensis*, *N*. *medogensis*, and *N*. *nyalamensis*) have only sequence from mitochondrial loci available. However, well supported sister taxa relationships were recovered between *N*. *clarkei* and *N*. *medogensis*, *N*. *leucurus* and *N*. *fuscus*, and *N*. *irene* and *N*. *linzhiensis*. Our analyses support the need for both mitochondrial and nuclear loci to resolve species relationships among arvicolines, as addition of just a few nuclear loci markedly improved the support values.

### Molar occlusal patterns

**Fig 6:**
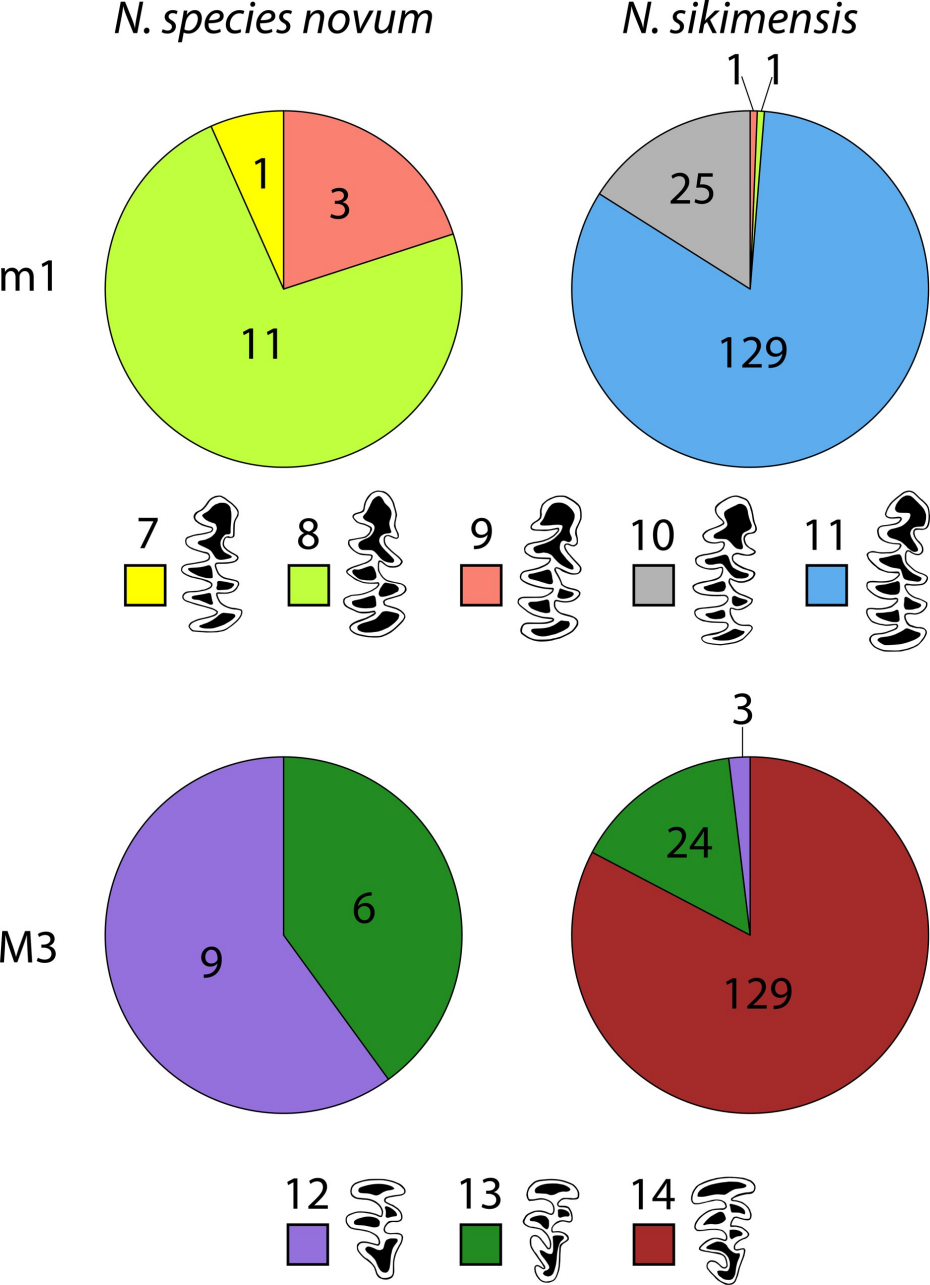
Pie chart of variation of molar occlusal patterns. Comparison of first lower molar (m1) and third upper molar (M3) of *Neodon species novum* (n = 15) and *Neodon sikimensis* (n = 156).

Among the samples of *Neodon* from western Nepal for which sequence data were obtained, three specimens were vouchered (Table 1). These three *N*. *species novum* had molar occlusal pattern (S8 Fig) more complex than observed in *N*. *irene* and less complex than observed in specimens of *N*. *sikimensis* from Sikkim, reported from Tibet [20] and observed in most specimens of *Neodon* from eastern Nepal. The molar patterns were most similar with those observed in other specimens of *Neodon* from western Nepal. We combined all of the *Neodon* from western Nepal into *N*. *species novum* based on their simpler m1 and M3 occlusal patterns (S9 Fig). Population sampled from western Nepal, *N*. *species novum*, and *N*. *sikimensis* from eastern Nepal and Sikkim have marked differences in the predominant occlusal patterns of their m1 and M3 molars (Fig 6).

### Morphometrics

*Neodon species novum* was significantly smaller than *N*. *sikimensis* in four cranial, three dental and three external measurements, but was significantly larger in breadth across the upper incisors. Compared with *N*. *irene*, *N*. *species novum* was significantly larger in all measurements examined other than ear height. Although *N*. *species novum* was significantly larger than *N*. *irene* in all measurements, the MANOVA failed to differentiate *N*. *nepalensis* and *N*. *irene* based on the non-significant Bonferroni-corrected *p*-value in the pairwise comparisons when all three species were included but yielded a highly significant *p*-value differentiating the two species when *N*. *sikimensis* was excluded. The pairwise comparisons conducted in PAST for MANOVA use within-group covariance matrix pooled over all groups, which could lead to discrepancies in *p*-values if groups are included/excluded.

In the two-dimensional plot in multivariate space *N*. *species novum* was more similar to *N*. *sikimensis*, though all three species were clearly differentiated. Measurements reported for *N*. *sikimensis* from Tibet included only mean, maximum, and minimum values [20] and without standard error or deviation being reported, limited statistical analyses could be conducted. A CVA including the mean, maximum, and minimum values of Tibet specimens [20] cluster with the *N*. *sikimensis* specimens.

All three species were clearly separated by canonical variance analysis and the discriminant analysis correctly identified over 97% of the specimens with only two specimens of *N*. *sikimensis* being identified as *N*. *species novum*. Specimens from western Nepal, *N*. *species novum*, are morphologically distinct from both *N*. *sikimensis* and *N*. *irene*.

### *Neodon sikimensis* Horsfield, 1851 –Sikkim Vole

One of the problems in trying to address the variation observed among *Neodon* occurring in Nepal is the lack of a good description of *N*. *sikimensis*. Not only is this taxon poorly described [7] but there is some confusion about the author and date of the limited description that is available. Kaneko and Smeenk [56] clarified that the author and date of the publication of the Sikkim vole *N*. *sikimensis* is Horsfield (1851) [1] rather than Hodgson (1849). The paper of 1849 was a letter published in the Annals and Magazine of Natural History written by Horsfield [57] commenting on a specimen that Hodgson considered to be a new species and pointing out that the molars had some unique feature that would be described in detail later by Hodgson. Hodgson’s expected description never appeared and Horsfield (1851:145–146) [1] formally described the genus *Neodon* and *N*. *sikimensis* based on characteristics of a single specimen from Sikkim. Kaneko and Smeenk [56] concluded that specimen BM 79.11.21.395 in the British Museum is the holotype and reported that there is no skull for this specimen.

Horsfield [1] described *N*. *sikimensis* as having very soft and silky uniform pelage that was deep brownish black, with a slight rusty shade dorsally, with the chin, breast, and abdomen being a deep bluish gray, with a slightly ferruginous shade. The ears were described as moderate in size and hairy externally. The tail was described as short (1½ inches) and a head and body of 5 inches. Jerdon [58] provided a similar description of the external morphology and reported measurements of the head and body as 4¾ inches, tail length 1½ inches, and hind foot ¾ of an inch of an individual collected in Sikkim, near Darjeeling.

Three specimens on *N*. *sikimensis* all collected by B. H. Hodgson from Sikkim were donated to the British Museum of Natural History in 1879 by the Museum of the East-India Company (P. Jenkins, pers. com.). In addition to the holotype (1879.11.21.395) specimen 1879.11.21.397 (skin only) and 1879.11.21.396 (skin with a damaged skull) were collected from the type locality. Specimen 1879.11.21.396 displays complex occlusal patterns of the m1 and M3 (S10 Fig) characteristic of the descriptions provided by Hinton [3] and Agrawal [59] with an anterior loop followed by two pairs of confluent triangles, three closed triangles and a posterior loop in the m1 (pattern 11, Fig 2) while the M3 possesses a long posterior loop with a well-developed third inner angle (pattern 14, Fig 2). Variation in the occlusal patterns of the m1 and M3 has been reported [16] and variation within and between populations was observed in our examination of additional specimens (S9 Fig). In general, specimens from eastern Nepal, had more complex m1 and M3 occlusal patterns characteristic of *N*. *sikimensis* from Sikkim and Tibet.

The Sikkim vole has been characterized by its large size (hind foot 20 mm and condyle-basal length of skull 28 mm) [3] and more complex cheek teeth [3, 59]. Eight cranial characters were examined from a single specimen collected in 1915 from Lachen, Sikkim [3] and standard external and six cranial characters were examined from 12 and 16 specimens from India [59], respectively. In addition, standard external and 11cranial characters were examined in 13–16 specimens from Tibet [20]. Unfortunately, there is limited overlap in the cranial measurements reported among these three studies [3, 20, 59] but there would appear to be differences in several characters between populations sampled from Tibet and India. For example, the head and body to tail ratio is 2.14 for specimens from eastern Nepal and India compared to 2.55 for specimens from Tibet.

The holotype of *N*. *sikimensis* (BM 79.11.21.395) identifies the type locality only as Sikkim, India [1, 56]. Jerdon [58] stated “it [*N*. *sikimensis*] had only been procured in Sikkim, near Darjeeling, at heights varying from 7,000 to 15,000 feet.” Thus, we propose to restrict the type locality to Sikkim, near Darjeeling. The distribution of the Sikkim vole extends from eastern Nepal into northern India and southern Tibet [1, 16, 20, 30, 58] and has not been verified to occur in either Sichuan or Yunnan [27].

Together, the results from the DNA sequence data, the and molecular phylogenetic analyses, the geographic distribution of the variation in molar occlusal patterns, and the morphometric analyses supports the hypothesis that the individuals (previously referred to as *N*. *sikimensis*) from western Nepal represent an undescribed species of *Neodon*. Below, these populations of *Neodon* from western Nepal are formally described as a new species.

### *Neodon nepalensis*, species novum

#### Holotype

Field Museum of Natural History (FMNH) 142081; adult female; skin and skull (S11 Fig). Original number R. M. Mitchell 2112.

#### Type locality

Nepal, Dolpo District, Dhorpatan, 8,950 ft (UTM R44-703810-3159902; N 28^o^33’ E 83^o^05’), collected 3 February 1969.

#### Paratypes

Skins and skulls of four males (FMNH142080, FMNH142082, FMNH142083, NMNH142084 and one female FMNH142079) deposited in the Field Museum of Natural History.

#### Diagnosis

A species of *Neodon* with the following characteristics: size medium for the genus; tail about one-third the length of the head and body; hind foot medium; ear small; dorsal coloration dark (Buffy Brown at tips, Deep Mouse Gray at base, with guard hairs projecting 3–5 mm above pelage with lighter Cream Color tips; color nomenclature following Ridgeway [60]; sides Clay Color; venter Clay Color tips, Mouse Gray base, and lighter Cream Color middle band; tail bicolored, Buffy Brown above and Clay Color below; feet Cream Color with claws about 2.5 mm in length; ears Mummy brown; vibrissae black, 10–15 mm in length, some with white tips; cranium square, 1.7 times as long as wide; rostrum short, NL 85.6% of RL and RL 32.7% of SGL; molar tooth row 26.1% of SGL; incisors proodont; m1 with only one complete anterior confluent loop; M3 without or with very small third labial angle.

#### Distribution

West of the Trisuli River in western Nepal at elevations between 2400 and 4200 m. Recorded from Ghorepani (2800 m), Apoon Hill (3200 m), Tukche (~2800 m), Dhorpatani (~2800 m), Shey Gompa (~4200 m), Renchi (3030 m), Ringmo (3750 m), Jumla (2400 m), Mugu (~2900 m) and Rara Lake (2975 m).

#### Measurements

External measurements of the holotype as taken in the field (in mm) are: total length, 130.5; tail length, 31; hindfoot, 19.9; and ear, 12.7. Cranial measurements were obtained using dial calipers (in mm) and are as follows: SGL, 25.6; ZB, 14.8; IOW, 3.55; MB, 12.04; SH, 9.12; ABL, 6.79; LMxT, 6.69; LMbT, 6.19; MM, 5.46; and TUIB, 2.61. Mean measurements and standard errors for the five paratypes are as follows: TBL, 134±1.85; TL, 33.65±1.38; HF, 19.93±0.18; EL, 12.68±0.19; SGL, 25.9±0.17; ZB, 15.15±0.13; IOW, 3.78±0.08; MB, 11.92±0.06; SH, 9.11±0.06; ABL, 6.73±0.1; LMxT, 6.66±0.05; LMbT, 6.36±0.07; MM, 5.48±0.05; and TUIB, 2.74±0.12. Measurements of the three specimens in the private collection in Nepal are presented in S1 Table and the mean measurements and standard errors for these and additional specimens are presented in Table 6.

#### Comparisons

A species of *Neodon* resembling *N*. *sikimensis* and *N*. *irene* but is smaller than *N*. *sikimensis* and larger than *N*. *irene*. Tail shorter but with a higher head and body to tail ratio (2.99) than *N*. *sikimensis* (2.14–2.55) and longer than in *N*. *irene* but with lower head and body to tail ratio (3.13). Much smaller but with a longer tail than *N*. *leucurus* which has a head and body to tail ratio of 3.33 or greater. The ventral pelage of *N*. *nepalensis* is brown (Clay colored tips) compared with the lighter venters of *N*. *sikimensis* (Cream Buff tips) and *N*. *irene* (Slaty washed with gray white). *Neodon nepalensis* is significantly smaller than *N*. *sikimensis* in total body length, tail length, ear height, and 6 cranial measurements including greatest length of the skull, condylobasal length, mastoid breadth, skull height, length of the mandibular tooth row, width across molars but is significantly larger in breath of incisors. This newly described taxon is significantly larger than *N*. *irene* in all measurements other than ear height.

The molar patterns of the m1 and M3 observed in *N*. *nepalensis* are simpler than those observed in *N*. *sikimensis* and are more similar to those observed in *N*. *irene*. A m1 with an anterior loop followed by a single pair of confluent triangles, three alternating closed triangles, and a posterior loop resulting in four lingual and three labial folds is characteristic of *N*. *nepalensis* (Fig 2, pattern 8) though some individuals showed an unpaired addition confluent triangle following the anterior loop (Fig 2, pattern 9). The m1 of *N*. *sikimensis* contained two pairs of confluent triangles following the anterior loop (Fig 2, pattern11), though specimens with only one triangle of the second pair were observed (Fig 2, pattern 10). Specimens of *N*. *irene* examined also displayed a m1 with a single pair of confluent triangles (Fig 2, pattern 7) as has been previously reported [16, 19, 30]. The m1 of *N*. *sikimensis* contained five lingual and four labial folds whereas four lingual and three labial folds were observed of the m1 of *N*. *nepalensis* and *N*. *irene*.

A third upper molar with an anterior loop followed by two alternating closed triangles and an open triangle that was confluent with a posterior loop without a medially inflected end was characteristic of *N*. *nepalensis* (Fig 2, pattern 12), though some individuals had either a weakly defined medially inflected end of the posterior loop or an additoional medial triangle that was not closed (Fig 2, pattern 13). The more complex M3 of *N*. *sikimensis* had a clearly defined medially inflected end of the posterior loop (Fig 2, pattern 14, though this was weakly defined in some specimens (Fig 2, pattern 13). No medial inflection was observed of the posterior loop of the M3 of *N*. *irene* and this tooth had only 2 lingual folds (Fig 2, pattern 12). The M3 of *N*. *nepalensis* contained either two lingual folds or two with a hint of a third fold whereas *N*. *sikimensis* usually contained a M3 with three lingual folds and rarely two with a hint of a third.

*Neodon nepalensis* demonstrates differentiation from other species of *Neodon*, including *Neodon sikimensis* from Tibet in both of the mitochondrial genes examined (*Cytb* and *COI*). Although sequence data are not available from *N*. *sikimensis* from eastern Nepal or India, *N*. *nepalensis* is morphologically distinct from samples examined from those regions.

#### Habitat

Inhabits edges of rhododendron (*Rhododendron* sp.) forest, coniferous (*Abies* sp., *Cedrus* sp., *Picea* sp., *Pinus* sp.) forests, scrub habitat with peashrub (*Caragana* sp,) and honeysuckle (*Lonicera* sp.) and ecotones between scrub and forest at elevations between 2400 and 4200 m. Also reported from areas with clumps of ephedra (*Ephedra* sp.) and stunted junipers (*Juniperus* sp.) [61]. Restricted to the western Himalayan broadleaf and coniferous forest, whereas *N*. *sikimensis* occurs in the eastern Himalayan broadleaf and coniferous forest in Nepal.

#### Remarks

Results from the phylogenetic, genetic distance and morphological analyses support the recognition of *Neodon nepalensis* as a distinct species under phylogenetic, genetic and morpho species concepts.

#### Etymology

This species is named to recognize its endemic distribution to Nepal. The common name Nepalese vole is recommended.

#### Nomenclatural statement

A life science identifier (LSID) number was obtained for the new species *Neodon nepalensis*: urn:lsid:zoobank.org:act: 881FFC1FF-4Ø2Ø-4533-B98D-E93C8ØC4FC22.

#### Specimens examined

Additional material, including specimens examined, are identified in S3 Appendix.

### Other potentially unidentified cryptic species of *Neodon* in Nepal

Genetic distances between *N*. *nepalensis* sampled from Myagdi District (GPN16 and GPN25) and Dolpo District (RCH4, RGM4, RGM6, and RGM11) were between 7.6 to 8.0, which are comparable to the reported mean between sister species of *Microtus* [41]. The topography between these regions where these specimens were collected in western Nepal are separated by deep river valleys created by the Kali Gandaki River and numerous high mountains which may act as barriers to gene flow. However, the current sampling represents two populations without any intermediate sampling of localities and the divergence observed may be an effect of isolation by distance. The current sampling does not provide data to exclude either of these alternative hypotheses. Hence, more intensive sampling of *N*. *nepalensis* is need, especially in the areas between the localities from which sequence data were obtained, to determine if additional cryptic species of *Neodon* occur in western Nepal.

Specimens (BMNH 1922.3.2.20 and 1922.3.2.21) collected in 1921 from East Everest at 17,000 ft in elevation were recognized as *Phaiomys everesti* [62] and the type locality for this taxon has been reported as northern Nepal [7, 63]. The original description of *P*. *everesti* [62] indicated that it resembled *P*. *waltoni* but was considerable smaller and *P*. *waltoni* [64] was described as smaller than *P*. *blythi*. Since its description [62] this small taxon has been considered as a subspecies of *Microtus* (*Neodon*) *leucurus* [7], a synonym of *M*. *leucurus waltoni* [65], a synonym of *M*. *leucurus* [11], or a synonym of *P*. *leucurus* [7]. *Phaiomys leucurus* now recognized as *N*. *leucurus* [20] has also been reported to occur in the Mustang District of northcentral Nepal [26, 61, 66]. A few specimens examined, currently recognized as *N*. *sikimensis* from northeastern Nepal (Solukhumbu and Sankhuwasabha Districts) collected by the Arun Valley Wildlife Expedition (1972–1973) and by Mitchell [61], have simple molar patterns (m1 and M3) not characteristic of the Sikkim vole (S9 Fig). The localities from which these voles with simpler molars were collected include Khumjung (FMNH 142092–1420940 and Num (FMNH 114333 and 114336), sites approximately 10 mi SW and 35 mi SE of the type locality of *P*. *everesti*, respectively. Additional research is needed to determine if a population of *Neodon* occurring in northeastern Nepal near Mount Everest represent a population of *N*. *leucurus* or if this material represents an additional cryptic species.

## Supporting information

S1 Appendix Sequences used in phylogenetic analyses. GenBank accession numbers of mitochondrial (*Cytb*, *COI*) and nuclear (*Ghr*, *Rbp3*) genes of select taxa of Arvicolinae and outgroup taxa included in this study.
(PDF)

S2 Appendix *Neodon* Cytochrome *b* sequences. GenBank accession numbers of sequence used to calculate K2P genetic distances.
(PDF)

S3 Appendix Material of *N*. *species novum*, *N*. *sikimensis* and *N*. *irene* examined. Museums designation: PC = private collection Nelish Pradhan, FMNH = Field Museum of Natural History, ZFMK = Zoological Research Museum Alexander Koenig, SAF = Sichuan Academy of Forestry, SNU = Sichuan Normal University, BMNH = British Museum of Natural History, GNHM = Guangxi Natural History Museum. Other appreciations: ‡ = specimen examined, ‡md = specimen with missing measurements and excluded from multivariant analyses, A = molar patterns shown in Fig 2 in Liu et al. [20], B = mean and range of measurements including these specimens provided by Liu et al. [20].
(PDF)

S1 Table Measurements of three voucher specimen in collection of Nelish Pradhan. SGL = skull greatest length, SBL = skull basal length, CBL = condylobasal length, ZB = zygomatic breadth, MB = mastoid breadth, IOW = least interorbital width, SH = skull height, ABL = auditory bullae length, LMxT = length of maxillary tooth row, LMbT = length of mandibular tooth row, MM = width across upper molars, TUIB = breath across upper incisors, TBL = total body length, TL = tail length, HF = length of hind foot, and EL = length of ear.
(PDF)

S1 Fig Molar types. *Neodon* specimens from Nepal examined by Nadachowski and Zagorodnyuk [16] to illustrate the variation in lower (m1: 7–11) and upper (M3: 12–14) molar. ZFMK 84.347 –Tukche; ZFMK 84.918 –Dhorpatan; ZFMK 84.905 –Thakkhola, Tukche; ZFMK 84.910 –Khumbu, Phulung; ZFMK 84.915 –Thodung, Ramechap.
(TIF)

S2 Fig Bayesian majority rule consensus tree for Arvicolinae and outgroup taxa based on concatenated mitochondrial loci (*Cytb* and *COI*). Nodal support provided as Bayesian posterior probability values.
(TIF)

S3 Fig Bayesian majority rule consensus tree for Arvicolinae and outgroup taxa based on concatenated nuclear loci (*Rbp3* and *Ghr*). Nodal support provided as Bayesian posterior probability values.
(TIF)

S4 Fig Bayesian majority rule consensus tree for Arvicolinae and outgroup taxa based on *Cytb* gene. Nodal support provided as Bayesian posterior probability values.
(TIF)

S5 Fig Bayesian majority rule consensus tree for Arvicolinae and outgroup taxa based on *COI* gene. Nodal support provided as Bayesian posterior probability values.
(TIF)

S6 Fig Bayesian majority rule consensus tree for Arvicolinae and outgroup taxa based on *Rbp3* gene. Nodal support provided as Bayesian posterior probability values.
(TIF)

S7 Fig Bayesian majority rule consensus tree for Arvicolinae and outgroup taxa based on *Ghr* gene. Nodal support provided as Bayesian posterior probability values.
(TIF)

S8 Fig Photos of skulls and mandibles showing molar occlusal patterns of three voucher specimen in collection of Nelish Pradhan. (TIF)

S9 Fig Localities examined and observed molar patterns (m1/M3) in *Neodon species novum* (red) and *N*. *sikimensis* (yellow). **1.** Rara Lake, Mugu (8/12); **2.** Maharigaon, Jumla (8/12, 8/13); **3.** Ringmo, Dolpa (9/12); **4.** Dhorpatan, Dolpa (7/12, 8/12, 8/13); **5.** Ghorepani, Myagdi (9/12, 9/13); **6.** Phulung Ghyang, Nawakot (11/13, 11/14); **7.** Gosenkunde, Nawakot (11/13, 11/14); **8.** Langtang Village, Rasuwa (11/13); **9.** Uring Ghyang, Sindhu (10/14); **10.** Dhukphu, Sindhu (10/13); **11.** Tserping, Ramechap (11/14); **12.** Thodung, Ramechap (11/14); **13.** Lukla Airport, Solukhumbu (10/14, 11/14); **14.** Khumjung, Solukhumbu (8/12, 10/12); **15.** Kasua Khola, Sankhuwasabha (11/14); **16.** Num, Sankhuwasabha (10/13, 10/14, 11/14); **17.** Balutar, Sankhuwasabha (9/14, 10/14, 11/14); **18.** Chainpur, Sankhuwasabha (10/14); **19.** Jamnagaon, Ilam(11/14); **20.** Thangu, Sikkim (11/13, 11/14); **21.** Lachen, Sikkim (11/13, 11/14); **22.** Tibet (11/14 see Fig 2 [20]).
(TIF)

S10 Fig Upper and lower molars of *Neodon sikimensis*. Paratype specimen NHMUK 1879.11.21.39 from Sikkim.
(TIF)

S11 Fig Skull, mandible, and skin of holotype FMNH 142081. (TIF)
