# Supplementary material for: Further assessment of the Genus Neodon and the description of a new species from Nepal
Source: PLoS One. 2019 Jul 17;14(7):e0219157. doi: 10.1371/journal.pone.0219157 (PMC6636723; doi:10.1371/journal.pone.0219157)
Supplement: S1 Appendix — GenBank accession numbers of mitochondrial (Cytb, COI) and nuclear (Ghr, Rbp3) genes of select taxa of Arvicolinae and outgroup taxa included in this study. (PDF) [file pone.0219157.s001.pdf]

Genus	Species	Accession No.			
		Cytb	***COI***	***Ghr***	***Rbp3***
Mesocricetus	*M. auratus*	AF119265	JF444326	AF540632	FM162052
Arvicola	*A. terrestris*	AF159400	AY332681	KX455563	AY277407
	A. sapidus	FJ539341			JX457665
	A. scherman	JX457750			JX457670
Chionomys	*C. nivalis*	AY513845	AY332686	KP057338	KP057391
Lasiopodomys	*L. brandtii* 1	GQ352472	KF182204	GQ142008	JF906134
	L. brandtii 2	JF906120	KF182207	GQ374498	
	L. gregalis 1	KF839592	KP190315	GQ142007	
	L. gregalis 2	KF839591	KP190314		
	L. mandarinus 1	AM392373		AM392396	AM919413
	L. mandarinus 2	KF819832	KF819832	KP057333	KP057386
Alexandromys	*A. fortis*	KJ081954	HM137730	GQ374494	JF906129
	A. kikuchii	AF348082	AF348082	AM392385	AM919410
	A. limnoiphilus	HQ123615	JX962280		AM919426
	A. oeconomus	AY220032	KF152999	GQ374499	AM919418
Microtus	*M. chrotorrhinus*	AF163893		KX455572	KX455518
	M. agrestis	AY167213	JF499313	KX455568	KX455514
	M. afghanus 1	EF599109		KX455566	KX455512
	M. afghanus 2	EF599108			
	M. bucharensis 1	AM392369		AM392392	
	M. bucharensis 2	EF599110		KX455567	KX455513
	M. juldaschi 1	EF599113		KX455571	KX455517
	M. juldaschi 2	EF599112			
	M. juldaschi 3	AY513808			
	M. guatemalensis	AF410262	JN311722		
	M. socialis	KC953626	KF152993	FM162073	FM162055
	M. irani	FJ767748		KX455576	KX455522
	M. levis	NC_008064	NC_008064	KX455588	KX455532
	M. paradoxus	KC953624			
	M. transcaspicus	KX581066		AM910795	AM919405
	M. pennsylvanicus	KC473495	JN311723	AF540633	AM919415
	M. townsendii	AF163906			
	M. ochrogaster	DQ432006	JQ601444	KX455584	KX455529
	M. pinetorum	AF163904		MF074898	MF097761
	M. bavaricus	DQ841693			
	M. liechtensteini	EF379100			
	M. majori	AY513814		AM910796	AM919409
	M. schelkovnikovi	AM910619		AM910794	AM919408
Neodon	*N. sikimensis* 1	JF906124		GQ374496	
	N. sikimensis 2	HQ123606	KP190269		
	N. sikimensis 3	HQ123604	KP190272		

	N. sikimensis 4	HQ123603	KP190271		
	N. sikimensis 5	HQ123600	KP190266		
	N. sikimensis 6	HQ123601	KP190267		
	N. sikimensis 7	KU891252	KU891252		
	N. sikimensis 8	HQ123605	KP190270		
	N. sikimensis 9	HQ123599			
	N.irene 1	JF906127		GQ374493	JF906136
	N.irene 2	AM392370		AM392393	AM919412
	N.irene 3	HQ123596	KP190273		
	N.irene 4	HQ123619	KP190275		
	N.irene 5	HQ416908	HQ416908		
	N.irene 6			AY294924	AY163593*
	N.irene 7		KC709680		
	N.irene 8		JX962253		
	N.irene 9	GU908290			
	N.irene 10		HQ318710		
	N.irene 11			GU908395	
	N. irene 12	AY641526			
	N. leucurus 1	KP190225	KP190291		
	N. leucurus 2	KP190226	KP190292		
	N. clarkei 2	KP19022	KP190287		
	N. clarkei 3	KP190221			
	N. clarkei 4	KP190219	KP190286		
	N. linzhiensis 1	HQ123617	KP190262		
	N. linzhiensis 2	HQ123593	KP190264		
	N. nyalamensis 1	KP190227	KP190293		
	N. nyalamensis 2	KP190228	KP190294		
	N. medogensis 1	KP190214	KP190281		
	N. medogensis 2	KP190215	KP190282		
	N. fuscus 1	JF906122		GQ374495	JF906131
	N. fuscus 2	HQ123609	KP190276		
	N. fuscus 3	KP190210	KP190277		
	N. fuscus 4	KP190213	KP190280		
	N. fuscus 5	MG833880	MG833880		
	N. fuscus 6	KU214739			
	N. fuscus 7		JX962263		
	N. fuscus 8		JX962265		
	N. fuscus 9	AM392371		AM392394	
	N. sp. AM919400				AM919400
Dicrostonyx	*D. torquatus*	AF119275		AM392381	
	D. groenlandicus	AF119268	JF456464		KJ556766
Ellobius	*E. tancrei*	AF119270		GQ142006	
Lagurus	*L. lagurus*	AF429818	KF152989	GQ142003	

Lemmus	*L. sibricus*	AY219140		AM392398	AM919402
Myodes	*M. gapperi*	KJ789561	JF456940	AF540623	AY326080
	M. rufocanus	KX082749	JF903284	GQ142002	KJ556761
	M. rutilus	KJ789597	HM165301	KC962291	KC962308
Eothenomys	*E. melanogaster*	AY426682	HM165325	AM392399	AY163583
	E. miletus	AY426686	HM165305		KJ556773
Alticola	*A. argentatus*	KJ556727		KC962274	KC962294
	A. semicanus	DQ845192		KC962285	KC962297
	A. lemminus	KJ556621		KC962282	KC962296
Ondatra	*O. zibethicus*	KC563206	JF456977	AY294925	KC953427
Prometheomys	*P. schaposchnikowi*	AM392372		AM392395	AM919406
Phenacomys	*P. intermedius*	AF119260	JF457089	AM392377	KC953438

*Sequence AY163593 reported in GenBank as from *Neodon sikimensis* but specimen from which it was derived USNM 449126 is recorded as *N. irene*; concatenated with sequence from specimen of *N. irene* from the same location
