# Supplementary material for: Further assessment of the Genus Neodon and the description of a new species from Nepal
Source: PLoS One. 2019 Jul 17;14(7):e0219157. doi: 10.1371/journal.pone.0219157 (PMC6636723; doi:10.1371/journal.pone.0219157)
Supplement: S2 Appendix — GenBank accession numbers of sequence used to calculate K2P genetic distances. (PDF) [file pone.0219157.s002.pdf]

Species	Accession Number
Neodon clarkei	KP190221
	KP190220
	KP190219
Neodon fuscus	KP190213
	KP190212
	KP190211
	KP190210
	KU214739
	KU214738
	KU214737
	KU214736
	KU214698
	KU214683
	KU214682
	KU214681
	KU214680
	KU214679
	HQ123609
	JF906122
Neodon irene	GU908338
	GU908337
	GU908336
	GU908335
	GU908334
	GU908333
	GU908332
	GU908331
	GU908330
	GU908329
	GU908328
	GU908327
	GU908326
	GU908325
	GU908324
	GU908323
	GU908322
	GU908321
	GU908320
	GU908319
	GU908318
	GU908317

GU908316
GU908315
GU908314
GU908313
GU908312
GU908311
GU908310
GU908309
GU908308
GU908307
GU908306
GU908305
GU908304
GU908303
GU908302
GU908301
GU908300
GU908299
GU908298
GU908297
GU908296
GU908295
GU908294
GU908293
GU908292
GU908291
GU908290
HQ123619
HQ123616
HQ123614
HQ123611
HQ123597
HQ123596
HQ123595
KU214724
KU214688
KU214685
KP190209
JF906127
KX354149
HQ416908
AM392370
KU214689

	KU214687
	KU214686
	KU214684
	NC_16055
Neodon leucurus	KX455506
	KP190226
	KP190225
	KP190224
	KP190223
	KP190222
Neodon linzhiensis	HQ123618
	HQ123617
	HQ123594
	HQ123593
Neodon medogensis	KP190218
	KP190217
	KP190216
	KP190215
	KP190214
Neodon nyalamensis	KP190231
	KP190230
	KP190229
	KP190228
	KP190227
Neodon sikimensis	HQ123606
	HQ123605
	HQ123604
	HQ123603
	HQ123602
	HQ123601
	HQ123600
	HQ123599
	JF906124
	KU891252
	NC_35503
