# Supplementary material for: Further assessment of the Genus Neodon and the description of a new species from Nepal
Source: PLoS One. 2019 Jul 17;14(7):e0219157. doi: 10.1371/journal.pone.0219157 (PMC6636723; doi:10.1371/journal.pone.0219157)
Supplement: S3 Appendix — Museums designation: PC = private collection Nelish Pradhan, FMNH = Field Museum of Natural History, ZFMK = Zoological Research Museum Alexander Koenig, SAF = Sichuan Academy of Forestry, SNU = Sichuan Normal University, BMNH = British Museum of Natural History, GNHM = Guangxi Natural History Museum. Other appreciations: ‡ = specimen examined, ‡md = specimen with missing measurements and excluded from multivariant analyses, A = molar patterns shown in Fig 2 in Liu et al. [20], B = mean and range of measurements including these specimens provided by Liu et al. [20]. (PDF) [file pone.0219157.s003.pdf]

Species	Locality	Voucher	Museum	Occlusal Pattern	Morpho	*Cytb*	*COI*	*Rbp3*	*Ghr*
N. species novum	Ghorepani, Myagdi	NP 24	PC	‡	‡	MH558123	MH558119	MH558114	MH569055
	Ghorepani, Myagdi	NP 26	PC	‡	‡	MH558124	MH558120	MH558115	MH569056
	Rechi, Dolpo	released				MH558125			MH569057
	Ringmo, Dolpo	released				MH558126		MH558116	MH569058
	Ringmo, Dolpo	released				MH558127	MH558121	MH558117	MH569059
	Ringmo, Dolpo	NP 44	PC	‡	‡	MH558128	MH558122	MH558118	MH569060
Paratype	Dhorpatan, Dolpo	142079	FMNH	‡	‡				
Paratype	Dhorpatan, Dolpo	142080	FMNH	‡	‡				
Type	Dhorpatan, Dolpo	142081	FMNH	‡	‡				
Paratype	Dhorpatan, Dolpo	142082	FMNH	‡	‡				
Paratype	Dhorpatan, Dolpo	142083	FMNH	‡	‡				
Paratype	Dhorpatan, Dolpo	142084	FMNH	‡	‡				
	10 mi NE Jumla	142085	FMNH	‡	‡md				
	10 mi NE Jumla	142086	FMNH	‡	‡				
	Rana Lake, Mugu	142087	FMNH	‡	‡				
	Rana Lake, Mugu	142088	FMNH	‡	‡md				
	Rana Lake, Mugu	142089	FMNH	‡	‡				
	Rana Lake, Mugu	142103	FMNH	‡	‡md				
	Tukche, Mustang	84.837	ZFMK	photo					
	Dorpatan, Dolpo	84.918	ZFMK	photo					
	Tukche, Mustang	84.905	ZFMK	photo					
	Tukche, Mustang	84.935	ZFMK	photo					
N. sikimensis	Tibet	XZRAP 0117	SAF			JF906124			
	Tibet, Linzhi	GB821003	SAF	A	B	HQ123606	KP190269		
	Tibet, Linzhi	GB818009	SAF	A	B	HQ123604	KP190272		
	Tibet, Linzhi	GB817006	SAF	A	B	HQ123603	KP190271		
	Tibet, Linzhi	GB803001	SAF	A	B	HQ123600	KP190266		
	Tibet, Linzhi	GB815001	SAF	A	B	HQ123601	KP190267		

	Tibet	Unknown	SNU			KU891252	KU891251		
	Tibet, Linzhi	GB820001	SAF	A	B	HQ123605	KP190270		
	Tibet, Linzhi	GB802001	SAF	A	B	HQ123599			
	Sikkim	35831	FMNH	‡	‡md				
	Sikkim	35832	FMNH	‡	‡				
	Sikkim	35833	FMNH	‡	‡md				
	Sikkim	35834	FMNH	‡	‡md				
	Sikkim	35835	FMNH	‡	‡				
	Sikkim	35836	FMNH	‡	‡				
	Sikkim	35837	FMNH	‡	‡				
	Sikkim	35838	FMNH	‡	‡md				
	Sikkim	35839	FMNH	‡	‡md				
	Sikkim	35841	FMNH	‡	‡				
	Sikkim	35843	FMNH	‡	‡				
	Sikkim	35844	FMNH	‡	‡				
	Sikkim	35845	FMNH	‡	‡				
	Sikkim	35846	FMNH	‡	‡				
	Sikkim	35847	FMNH	‡	‡				
	Sikkim	35848	FMNH	‡	‡md				
	Sikkim	259547	NMNH	‡	‡				
	Sikkim	259548	NMNH	‡	‡				
	Nepal, Nawakot	57741	FMNH	‡	‡md				
	Nepal, Nawakot	57742	FMNH	‡	‡md				
	Nepal, Nawakot	57743	FMNH	‡	‡md				
	Nepal, Nawakot	57745	FMNH	‡	‡md				
	Nepal, Nawakot	57746	FMNH	‡	‡md				
	Nepal, Nawakot	57748	FMNH	‡	‡md				
	Nepal, Nawakot	57749	FMNH	‡	‡md				
	Nepal, Nawakot	57757	FMNH	‡	‡md				
	Nepal, Nawakot	57764	FMNH	‡	‡md				
	Nepal, Nawakot	104292	FMNH	‡	‡				
	Nepal, Nawakot	104293	FMNH	‡	‡md				
	Nepal, Nawakot	104294	FMNH	‡	‡				

	Nepal, Nawakot	104295	FMNH	‡	‡md				
	Nepal, Nawakot	104296	FMNH	‡	‡				
	Nepal, Nawakot	104297	FMNH	‡	‡				
	Nepal, Nawakot	104298	FMNH	‡	‡md				
	Nepal, Nawakot	104299	FMNH	‡	‡				
	Nepal, Nawakot	104301	FMNH	‡	‡				
	Nepal, Nawakot	104302	FMNH	‡	‡				
	Nepal, Nawakot	104303	FMNH	‡	‡				
	Nepal, Nawakot	104304	FMNH	‡	‡md				
	Nepal, Nawakot	104305	FMNH	‡	‡				
	Nepal, Nawakot	104306	FMNH	‡	‡				
	Nepal, Nawakot	104307	FMNH	‡	‡md				
	Nepal, Nawakot	104308	FMNH	‡	‡md				
	Nepal, Nawakot	104311	FMNH	‡	‡				
	Nepal, Nawakot	104313	FMNH	‡	‡				
	Nepal, Nawakot	104314	FMNH	‡	‡md				
	Nepal, Nawakot	104315	FMNH	‡	‡				
	Nepal, Nawakot	104316	FMNH	‡	‡				
	Nepal, Nawakot	104317	FMNH	‡	‡md				
	Nepal, Nawakot	104318	FMNH	‡	‡md				
	Nepal, Nawakot	104319	FMNH	‡	‡md				
	Nepal, Nawakot	104320	FMNH	‡	‡				
	Nepal, Nawakot	104321	FMNH	‡	‡				
	Nepal, Nawakot	104322	FMNH	‡	‡				
	Nepal, Nawakot	104323	FMNH	‡	‡				
	Nepal, Nawakot	104324	FMNH	‡	‡				
	Nepal, Nawakot	104325	FMNH	‡	‡				
	Nepal, Nawakot	104326	FMNH	‡	‡				
	Nepal, Nawakot	104327	FMNH	‡	‡				
	Nepal, Nawakot	104328	FMNH	‡	‡md				
	Nepal, Nawakot	104330	FMNH	‡	‡				
	Nepal, Nawakot	104331	FMNH	‡	‡				
	Nepal, Nawakot	104332	FMNH	‡	‡				

	Nepal, Nawakot	104333	FMNH	‡	‡md				
	Nepal, Nawakot	104334	FMNH	‡	‡				
	Nepal, Nawakot	104335	FMNH	‡	‡				
	Nepal, Nawakot	104336	FMNH	‡	‡				
	Nepal, Nawakot	104337	FMNH	‡	‡				
	Nepal, Nawakot	104338	FMNH	‡	‡				
	Nepal, Nawakot	104339	FMNH	‡	‡				
	Nepal, Nawakot	104340	FMNH	‡	‡				
	Nepal, Nawakot	104341	FMNH	‡	‡md				
	Nepal, Nawakot	104342	FMNH	‡	‡				
	Nepal, Nawakot	104343	FMNH	‡	‡md				
	Nepal, Nawakot	104344	FMNH	‡	‡md				
	Nepal, Nawakot	104347	FMNH	‡	‡				
	Nepal, Nawakot	104348	FMNH	‡	‡				
	Nepal, Nawakot	104349	FMNH	‡	‡md				
	Nepal, Nawakot	104350	FMNH	‡	‡md				
	Nepal, Nawakot	104351	FMNH	‡	‡				
	Nepal, Nawakot	104352	FMNH	‡	‡md				
	Nepal, Nawakot	104353	FMNH	‡	‡				
	Nepal, Nawakot	104354	FMNH	‡	‡md				
	Nepal, Nawakot	104355	FMNH	‡	‡				
	Nepal, Nawakot	104356	FMNH	‡	‡md				
	Nepal, Nawakot	104358	FMNH	‡	‡md				
	Nepal, Nawakot	104359	FMNH	‡	‡				
	Nepal, Nawakot	104360	FMNH	‡	‡md				
	Nepal, Nawakot	104361	FMNH	‡	‡				
	Nepal, Nawakot	104365	FMNH	‡	‡				
	Nepal, Nawakot	104368	FMNH	‡	‡				
	Nepal, Nawakot	104373	FMNH	‡	‡md				
	Nepal, Nawakot	104374	FMNH	‡	‡md				
	Nepal, Nawakot	104377	FMNH	‡	‡md				
	Nepal, Nawakot	104378	FMNH	‡	‡				
	Nepal, Ramechap	94162	FMNH	‡	‡				

	Nepal, Ramechap	94163	FMNH	‡	‡				
	Nepal, Ramechap	94164	FMNH	‡	‡md				
	Nepal, Ramechap	94165	FMNH	‡	‡				
	Nepal, Ramechap	94166	FMNH	‡	‡md				
	Nepal, Ramechap	142091	FMNH	‡	‡				
	Nepal, Ilam	94170	FMNH	‡	‡				
	Nepal, Sankhuwasabha	114185	FMNH	‡	‡				
	Nepal, Sankhuwasabha	114318	FMNH	‡	‡				
	Nepal, Sankhuwasabha	114319	FMNH	‡	‡				
	Nepal, Sankhuwasabha	114320	FMNH		‡md				
	Nepal, Sankhuwasabha	114321	FMNH	‡	‡				
	Nepal, Sankhuwasabha	114322	FMNH	‡	‡				
	Nepal, Sankhuwasabha	114323	FMNH	‡	‡				
	Nepal, Sankhuwasabha	114324	FMNH	‡	‡md				
	Nepal, Sankhuwasabha	114325	FMNH	‡	‡md				
	Nepal, Sankhuwasabha	114326	FMNH	‡	‡md				
	Nepal, Sankhuwasabha	114327	FMNH	‡	‡md				
	Nepal, Sankhuwasabha	114328	FMNH	‡	‡md				
	Nepal, Sankhuwasabha	114329	FMNH	‡	‡md				
	Nepal, Sankhuwasabha	114331	FMNH	‡	‡md				
	Nepal, Sankhuwasabha	114332	FMNH	‡	‡md				
	Nepal, Sankhuwasabha	114333	FMNH	‡	‡md				
	Nepal, Sankhuwasabha	114334	FMNH	‡	‡md				
	Nepal, Sankhuwasabha	114335	FMNH	‡	‡md				
	Nepal, Sankhuwasabha	114336	FMNH		‡md				
	Nepal, Sankhuwasabha	114337	FMNH	‡	‡md				
	Nepal, Sankhuwasabha	114338	FMNH	‡	‡md				
	Nepal, Sankhuwasabha	114339	FMNH	‡	‡md				
	Nepal, Sankhuwasabha	114340	FMNH	‡	‡md				
	Nepal, Sankhuwasabha	114595	FMNH	‡	‡md				
	Nepal, Sankhuwasabha	114596	FMNH	‡	‡md				
	Nepal, Sankhuwasabha	114598	FMNH	‡	‡md				
	Nepal, Sankhuwasabha	114602	FMNH	‡	‡md				

	Nepal, Sankhuwasabha	114603	FMNH	‡	‡md				
	Nepal, Sankhuwasabha	114605	FMNH	‡	‡md				
	Nepal, Sankhuwasabha	114606	FMNH	‡	‡md				
	Nepal, Sankhuwasabha	114610	FMNH	‡	‡md				
	Nepal, Sankhuwasabha	114611	FMNH	‡	‡md				
	Nepal, Sankhuwasabha	114614	FMNH	‡	‡md				
	Nepal, Sankhuwasabha	114615	FMNH	‡	‡md				
	Nepal, Sankhuwasabha	114616	FMNH	‡	‡md				
	Nepal, Sankhuwasabha	114617	FMNH	‡	‡md				
	Nepal, Sankhuwasabha	114618	FMNH	‡	‡md				
	Nepal, Sankhuwasabha	114620	FMNH	‡	‡md				
	Nepal, Sankhuwasabha	114621	FMNH	‡	‡md				
	Nepal, Sankhuwasabha	114623	FMNH	‡	‡md				
	Nepal, Sankhuwasabha	114624	FMNH	‡	‡md				
	Nepal, Sankhuwasabha	114625	FMNH	‡	‡md				
	Nepal, Sankhuwasabha	114628	FMNH	‡	‡md				
	Nepal, Rasuwa	142090	FMNH	‡	‡				
	Nepal, Solukhumbu	142092	FMNH	‡	‡				
	Nepal, Solukhumbu	142093	FMNH	‡	‡md				
	Nepal, Solukhumbu	142094	FMNH	‡	‡				
	Nepal, Solukhumbu	142095	FMNH	‡	‡				
	Nepal, Solukhumbu	142096	FMNH	‡	‡				
	Nepal, Solukhumbu	142097	FMNH	‡	‡				
	Nepal, Solukhumbu	142098	FMNH	‡	‡				
	Nepal, Solukhumbu	142099	FMNH	‡	‡md				
	Nepal, Solukhumbu	142100	FMNH	‡	Juvenile				
	Nepal, Solukhumbu	142101	FMNH	‡	‡				
	Nepal, Solukhumbu	142102	FMNH	‡	‡md				
	Nepal, Solukhumbu	142104	FMNH	‡	‡md				
	Nepal, Phulung	84.910	ZFMK	photo					
	Nepla, Ramechep	84.915	ZFMK	photo					
Paratype	Sikkim	1879.11.21.369	BMNH	photo					

N. irene	Unknown	SCDB003	GNHM			JF906127		JF06136	GQ374493
	Unknown	Unknown				AM392370		AM919412	AM392393
	Unknown	WL80					KC709680		
	Unknown	QH584-4					JX962253		
	Unknown	NIDB1							GU908395
	China, Yunnan	33939	FMNH	‡	‡md				
	China, Yunnan	33940	FMNH	‡	‡md				
	China, Yunnan	33941	FMNH	‡	‡md				
	China, Yunnan	33942	FMNH	‡	‡md				
	China, Yunnan	259931	NMNH	‡	‡md				
	China, Gansu	19084	FMNH		‡				
	China, Gansu	19085	FMNH		‡				
	China, Sichuan	07930	SAF			HQ123596	KP190273		
	China, Sichuan	06012	SAF			HQ123619	KP190275		
	China, Sichuan	Unknown				HQ416908	HQ416908		
	China, Sichuan	NIH1				GU908290			
	China, Sichuan	X84					HQ318710		
	China, Sichuan	240368	NMNH	‡	‡md				
	China, Qinghai	449167	NMNH		‡				
	China, Qinghai	449169	NMNH		‡				
	China, Qinghai	449170	NMNH		‡				
	China, Qinghai	449171	NMNH		‡				
	China, Qinghai	449172	NMNH		‡				
	China, Qinghai	449173	NMNH		‡			AY163593	AY294924
