# Supplementary material for: Further assessment of the Genus Neodon and the description of a new species from Nepal
Source: PLoS One. 2019 Jul 17;14(7):e0219157. doi: 10.1371/journal.pone.0219157 (PMC6636723; doi:10.1371/journal.pone.0219157)
Supplement: S1 Table — SGL = skull greatest length, SBL = skull basal length, CBL = condylobasal length, ZB = zygomatic breadth, MB = mastoid breadth, IOW = least interorbital width, SH = skull height, ABL = auditory bullae length, LMxT = length of maxillary tooth row, LMbT = length of mandibular tooth row, MM = width across upper molars, TUIB = breath across upper incisors, TBL = total body length, TL = tail length, HF = length of hind foot, and EL = length of ear. (PDF) [file pone.0219157.s004.pdf]

	Morphometric Characters							
**Specimen**	**SGL**	**SBL**	**CBL**	**ZB**	**IOW**	**MB**	**SH**	**ABL**
NP 24	24.67	23.39	24.73	15.6	3.66	11.82	9.42	6.03
NP 26	24.85	23.55	25.31	15.64	3.61	12.14	9.44	6.05
NP 44	25.07	24.0	25.75	15.48	3.76	11.47	8.85	6.18

	Morphometric Characters							
**Specimen**	**LMxT**	**LMbT**	**MM**	**TUB**	**TBL**	**TL**	**HF**	**EL**
NP 24	6.4	6.3	5.29	2.75	121	29	19	12
NP 26	6.66	6.34	5.44	2.73	132	31	17	12
NP 44	6.65	6.13	5.4	2.64	148	39	20	16
