# Supplementary figures and images for: Further assessment of the Genus Neodon and the description of a new species from Nepal
Source: PLoS One. 2019 Jul 17;14(7):e0219157. doi: 10.1371/journal.pone.0219157 (PMC6636723; doi:10.1371/journal.pone.0219157)

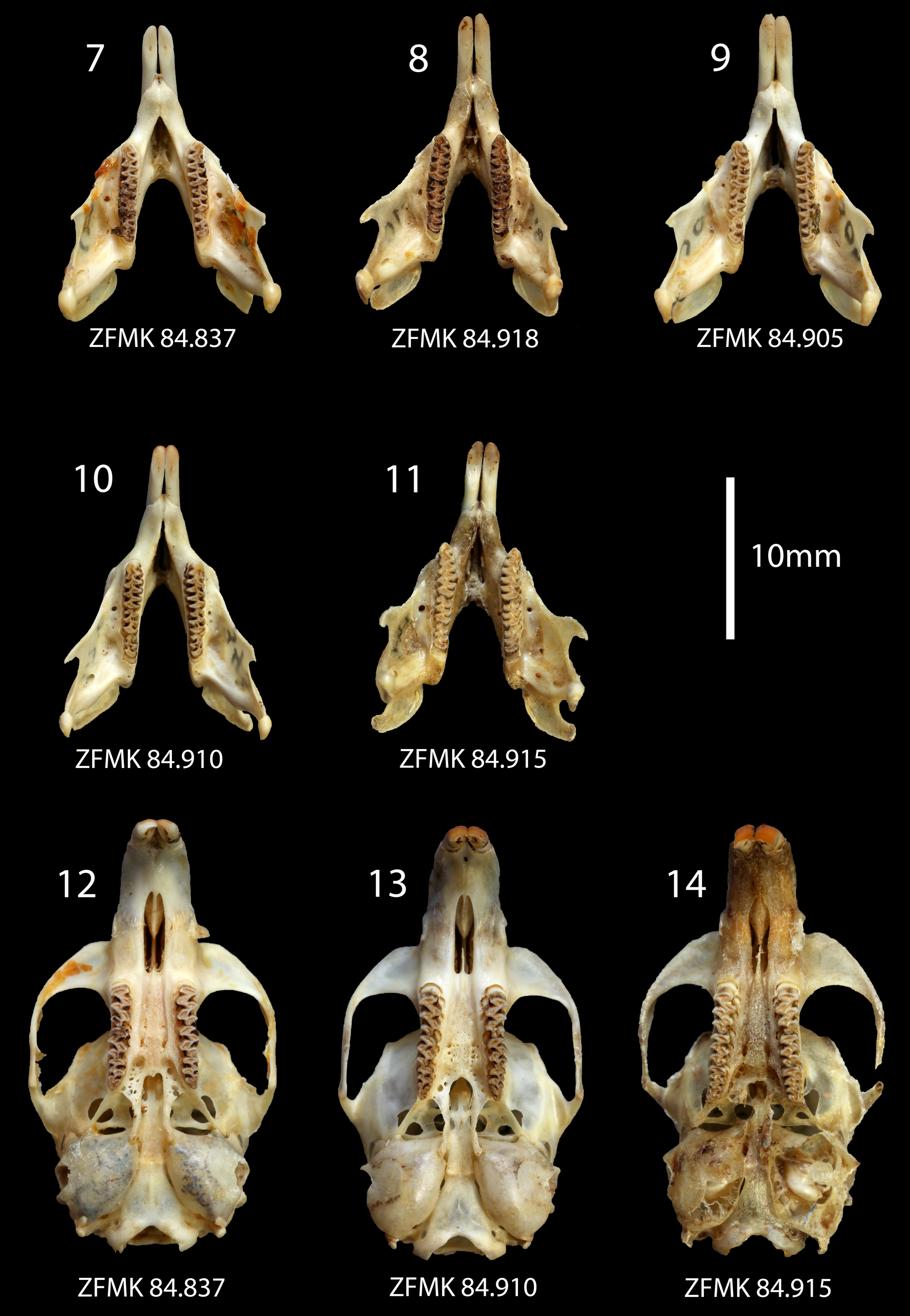

Supplement: S1 Fig — Neodon specimens from Nepal examined by Nadachowski and Zagorodnyuk [16] to illustrate the variation in lower (m1: 7–11) and upper (M3: 12–14) molar. ZFMK 84.347 –Tukche; ZFMK 84.918 –Dhorpatan; ZFMK 84.905 –Thakkhola, Tukche; ZFMK 84.910 –Khumbu, Phulung; ZFMK 84.915 –Thodung, Ramechap. (TIF) [file pone.0219157.s005.tif]

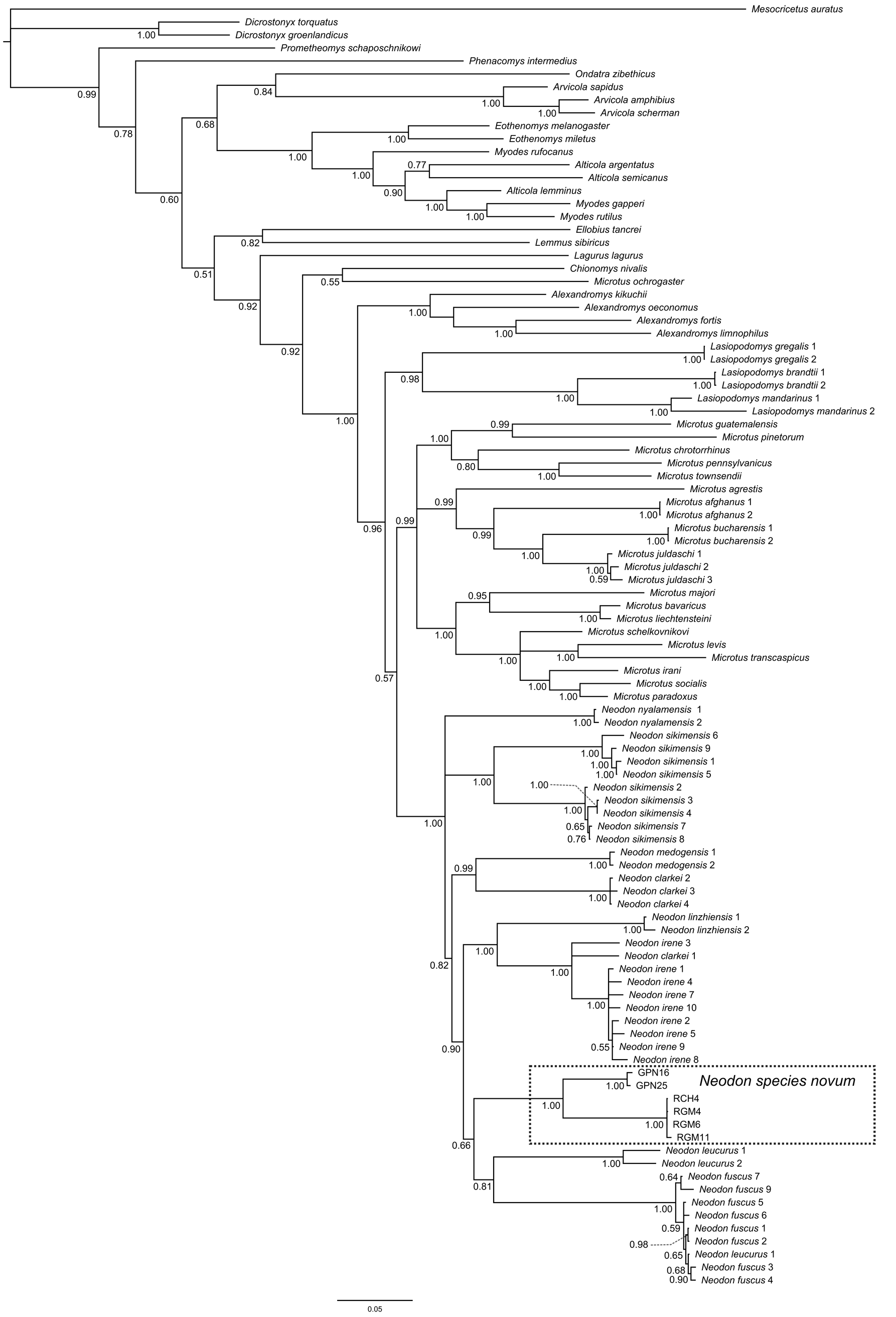

Supplement: S2 Fig — Nodal support provided as Bayesian posterior probability values. (TIF) [file pone.0219157.s006.tif]

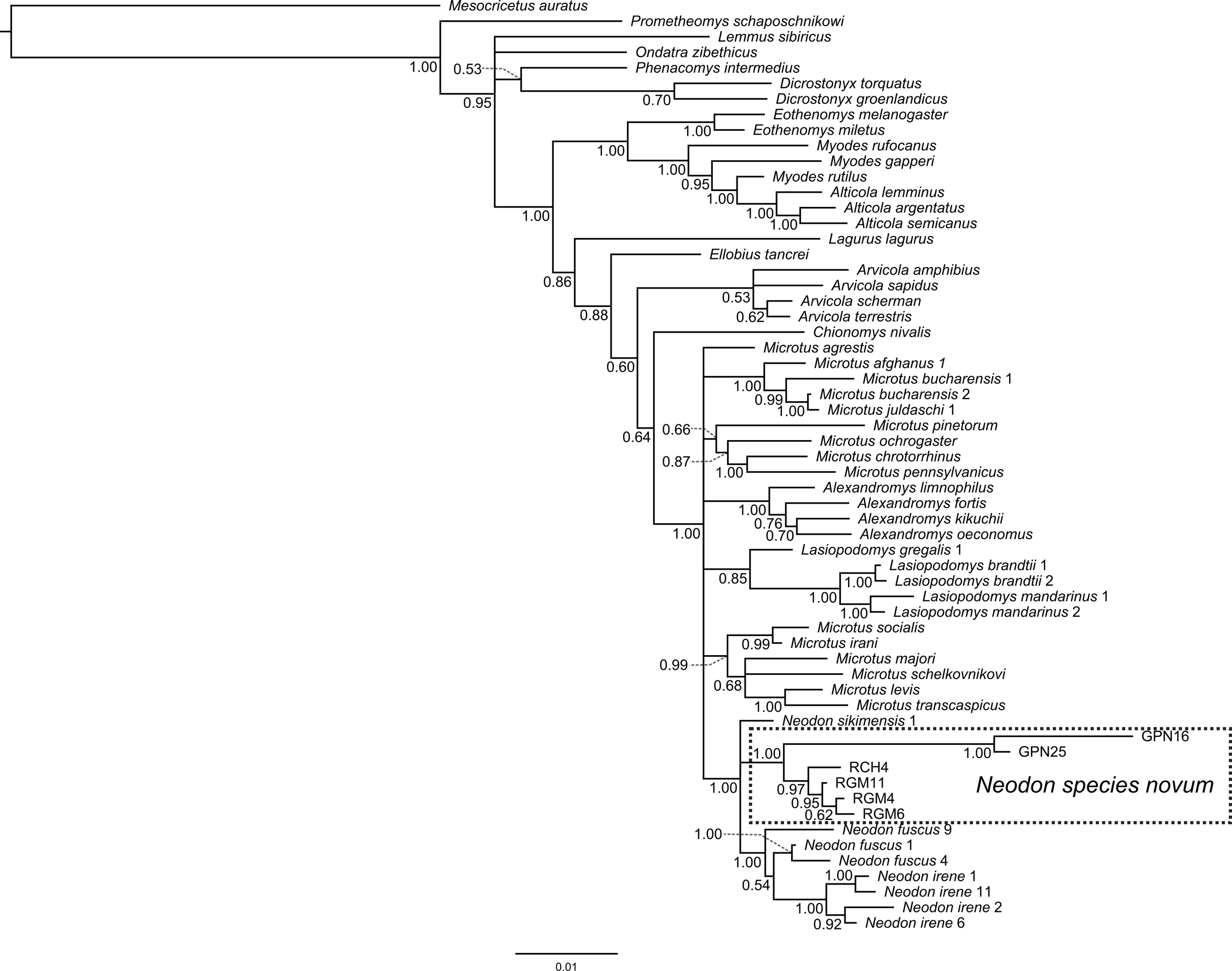

Supplement: S3 Fig — Nodal support provided as Bayesian posterior probability values. (TIF) [file pone.0219157.s007.tif]

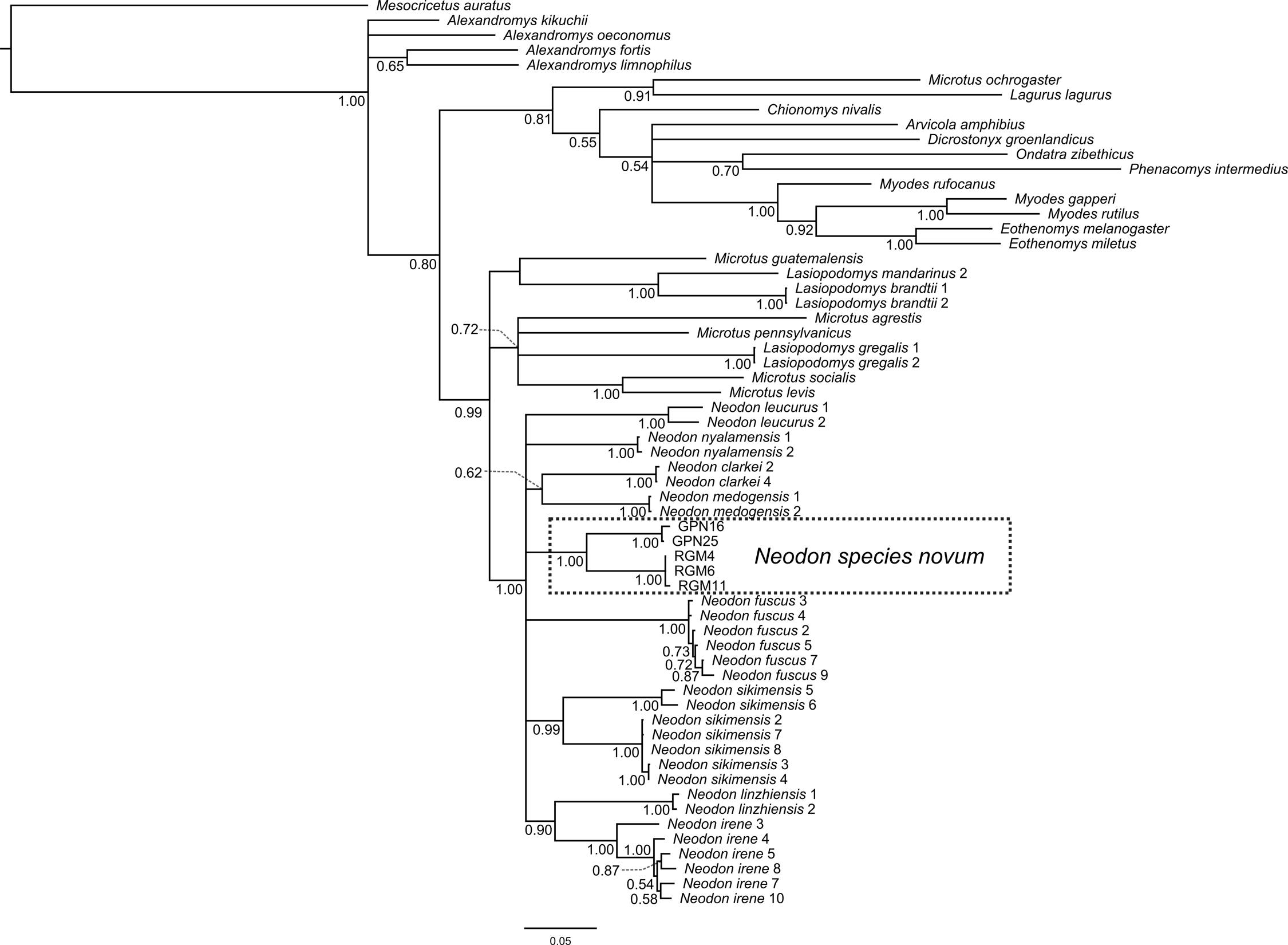

Supplement: S5 Fig — Nodal support provided as Bayesian posterior probability values. (TIF) [file pone.0219157.s009.tif]

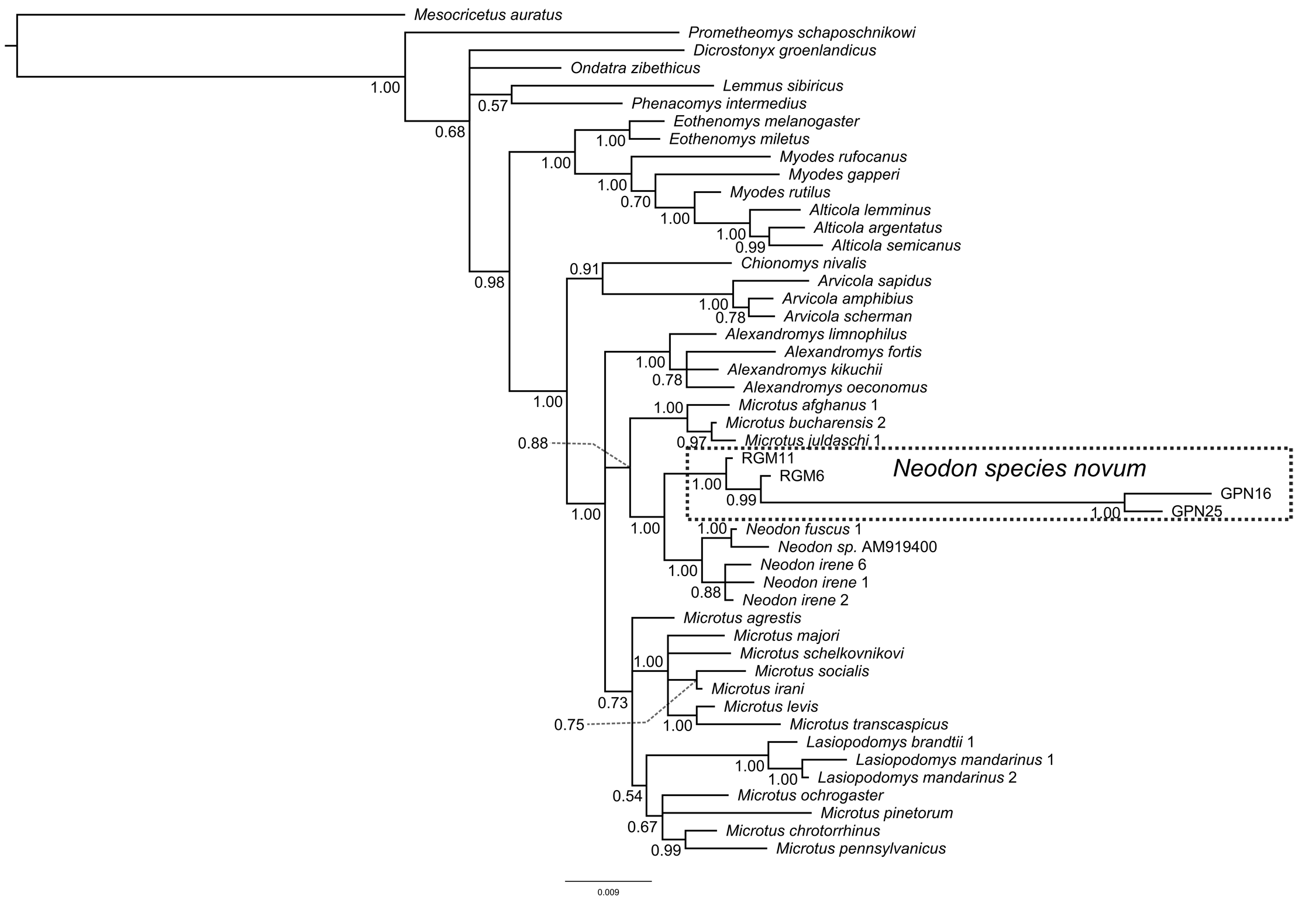

Supplement: S6 Fig — Nodal support provided as Bayesian posterior probability values. (TIF) [file pone.0219157.s010.tif]

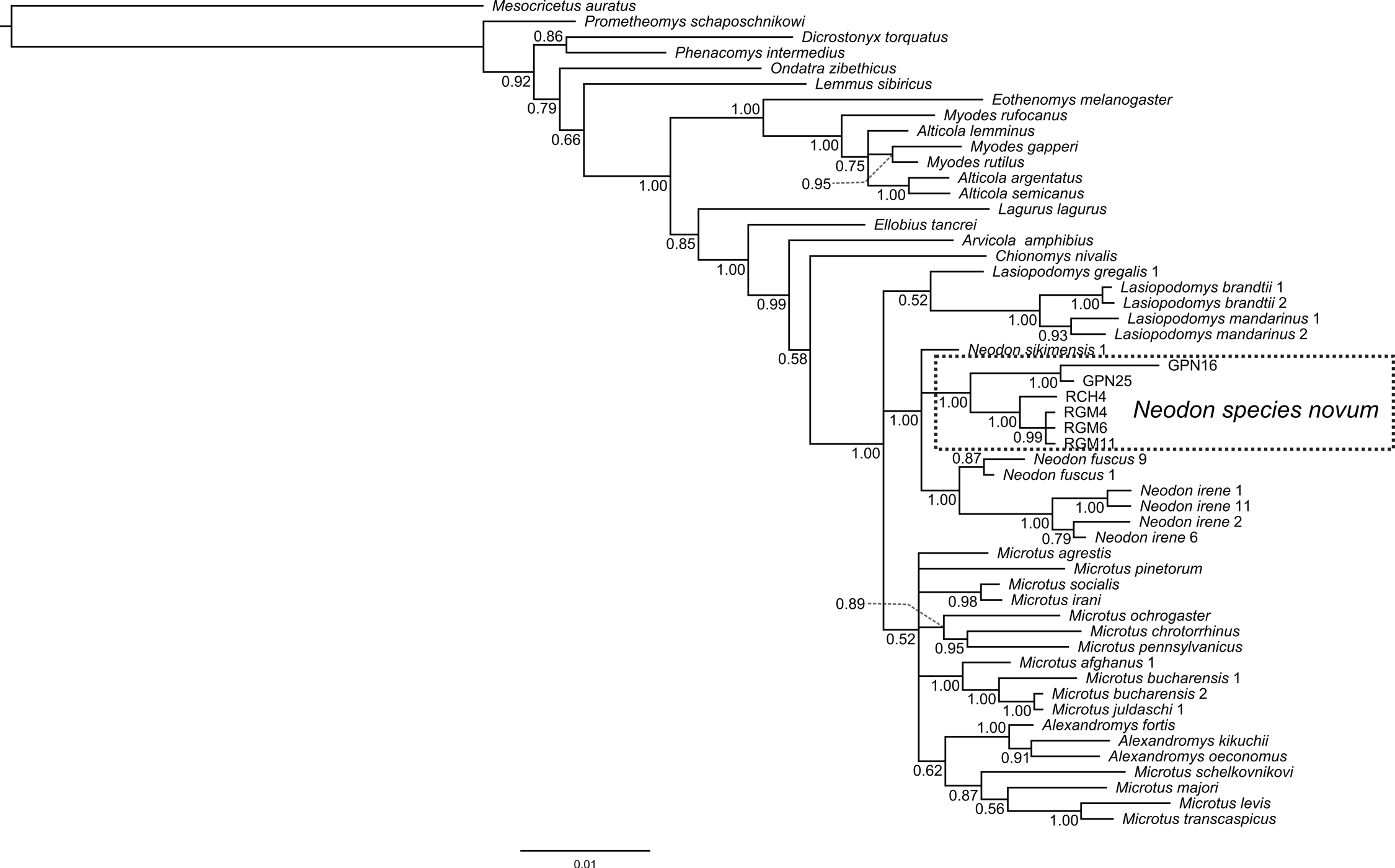

Supplement: S7 Fig — Nodal support provided as Bayesian posterior probability values. (TIF) [file pone.0219157.s011.tif]

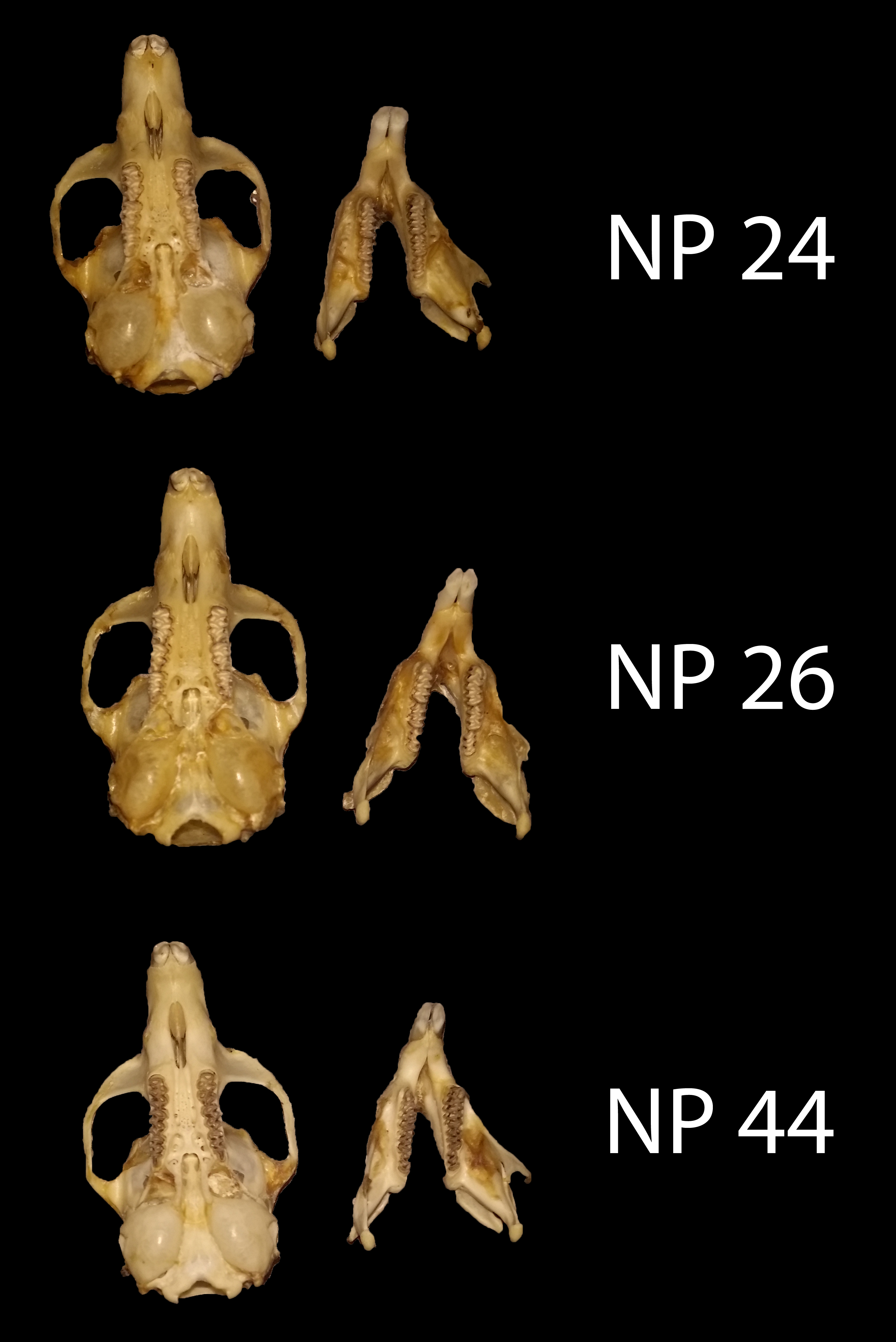

Supplement: S8 Fig — (TIF) [file pone.0219157.s012.tif]

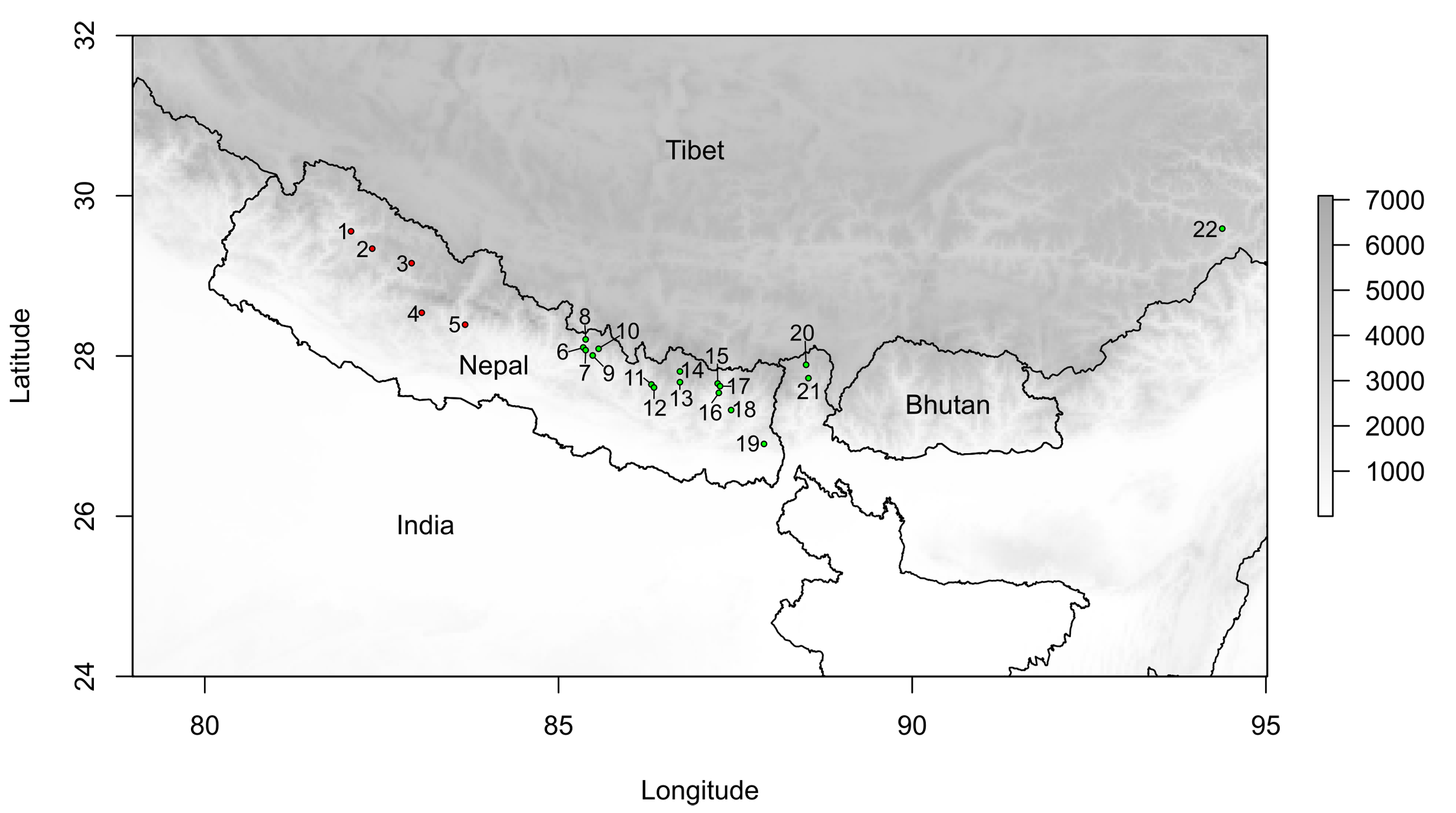

Supplement: S9 Fig — 1. Rara Lake, Mugu (8/12); 2. Maharigaon, Jumla (8/12, 8/13); 3. Ringmo, Dolpa (9/12); 4. Dhorpatan, Dolpa (7/12, 8/12, 8/13); 5. Ghorepani, Myagdi (9/12, 9/13); 6. Phulung Ghyang, Nawakot (11/13, 11/14); 7. Gosenkunde, Nawakot (11/13, 11/14); 8. Langtang Village, Rasuwa (11/13); 9. Uring Ghyang, Sindhu (10/14); 10. Dhukphu, Sindhu (10/13); 11. Tserping, Ramechap (11/14); 12. Thodung, Ramechap (11/14); 13. Lukla Airport, Solukhumbu (10/14, 11/14); 14. Khumjung, Solukhumbu (8/12, 10/12); 15. Kasua Khola, Sankhuwasabha (11/14); 16. Num, Sankhuwasabha (10/13, 10/14, 11/14); 17. Balutar, Sankhuwasabha (9/14, 10/14, 11/14); 18. Chainpur, Sankhuwasabha (10/14); 19. Jamnagaon, Ilam(11/14); 20. Thangu, Sikkim (11/13, 11/14); 21. Lachen, Sikkim (11/13, 11/14); 22. Tibet (11/14 see Fig 2 [20]). (TIF) [file pone.0219157.s013.tif]

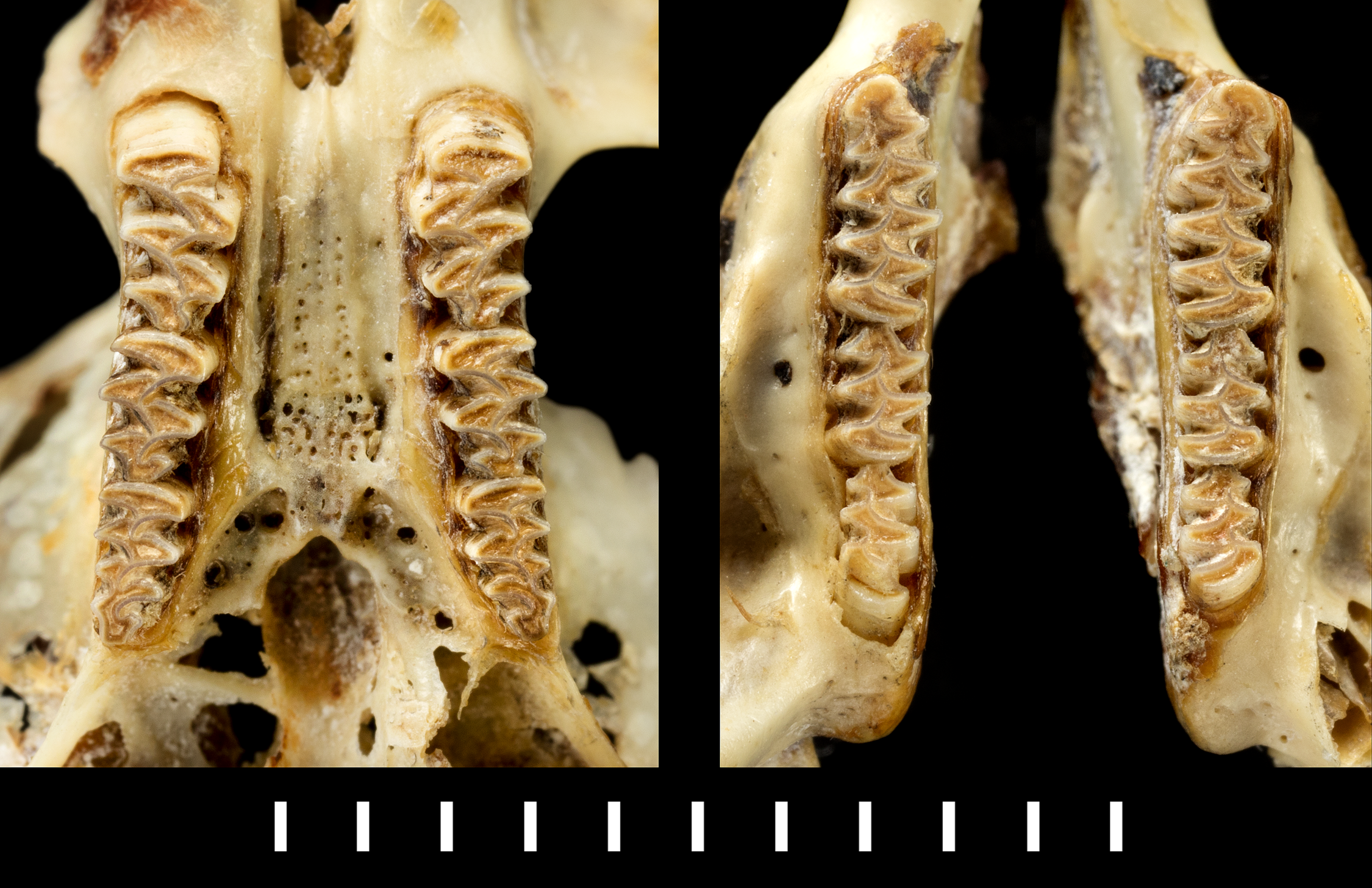

Supplement: S10 Fig — Paratype specimen NHMUK 1879.11.21.39 from Sikkim. (TIF) [file pone.0219157.s014.tif]

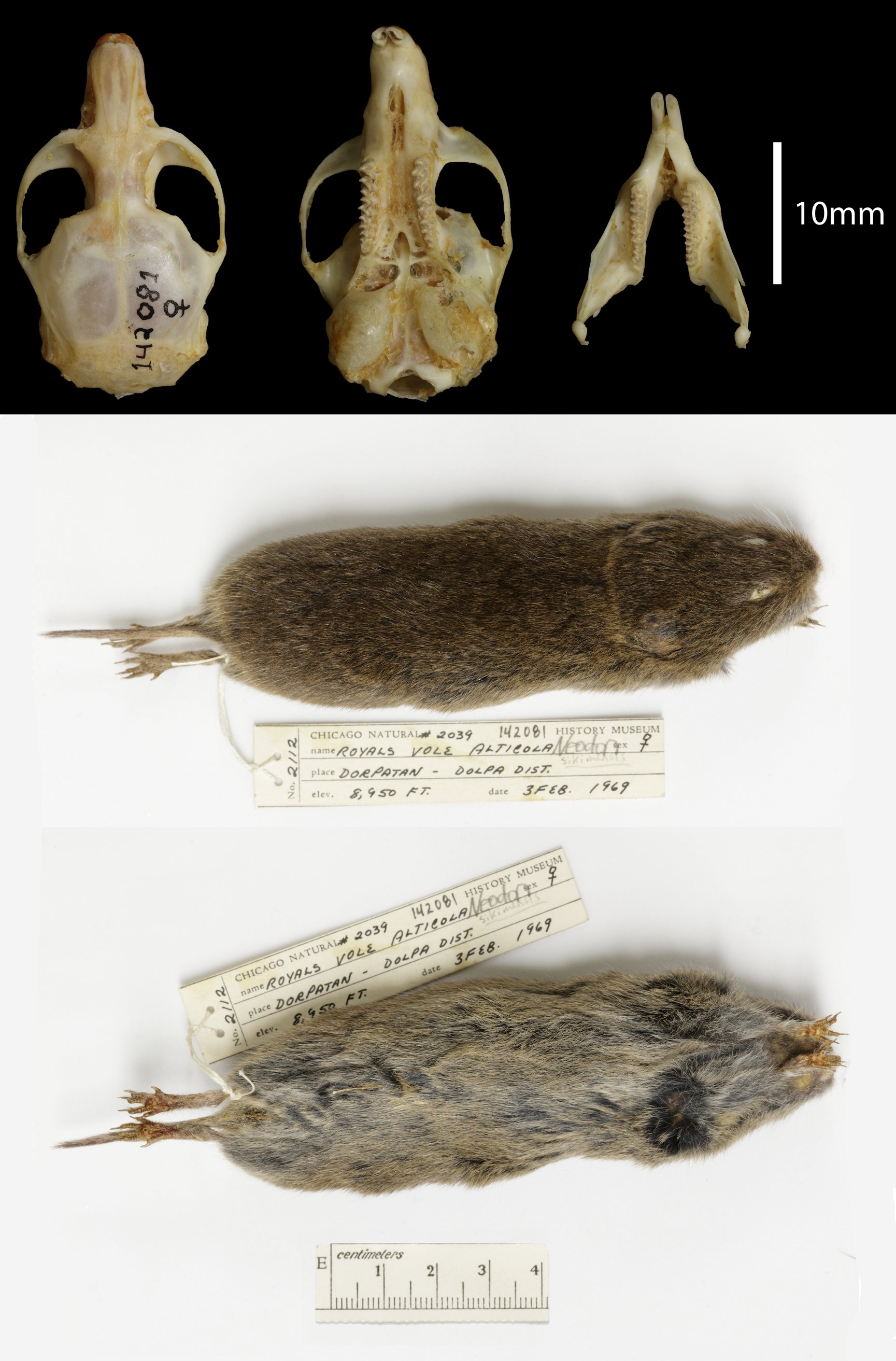

Supplement: S11 Fig — (TIF) [file pone.0219157.s015.tif]
